# A Novel Neuron-Specific Regulator of the V-ATPase in *Drosophila*

**DOI:** 10.1523/ENEURO.0193-21.2021

**Published:** 2021-10-21

**Authors:** Amina Dulac, Abdul-Raouf Issa, Jun Sun, Giorgio Matassi, Célia Jonas, Baya Chérif-Zahar, Daniel Cattaert, Serge Birman

**Affiliations:** 1 Genes Circuits Rhythms and Neuropathology, Brain Plasticity Unit, Unité Mixte de Recherche 8249, Centre National de la Recherche Scientifique, École Supérieure de Physique et de Chimie Industrielles de la Ville de Paris, Paris Sciences et Lettres University, Paris F-75005, France; 2 Dipartimento di Scienze Agroalimentari, Ambientali e Animali, University of Udine, Udine I-33100, Italy; 3 Université de Bordeaux, Centre National de la Recherche Scientifique, Institut de Neurosciences Cognitives et Intégratives d’Aquitaine, Unité mixte de Recherche 5287, F-33000 Bordeaux, France; 4 Centre National de la Recherche Scientifique, Unité Mixte de Recherche 7058 “Ecologie et Dynamique des Systèmes Anthropisés” (EDYSAN), Université de Picardie Jules Verne, Amiens cedex F-80025, France

**Keywords:** ATP6AP1L/ATP6AP1, CG31030/VhaAC45RP, *Drosophila melanogaster*, neuronal V-ATPase, quantal size, synaptic vesicle acidification

## Abstract

The V-ATPase is a highly conserved enzymatic complex that ensures appropriate levels of organelle acidification in virtually all eukaryotic cells. While the general mechanisms of this proton pump have been well studied, little is known about the specific regulations of neuronal V-ATPase. Here, we studied CG31030, a previously uncharacterized *Drosophila* protein predicted from its sequence homology to be part of the V-ATPase family. In contrast to its ortholog ATP6AP1/VhaAC45 which is ubiquitous, we observed that CG31030 expression is apparently restricted to all neurons, and using CRISPR/Cas9-mediated gene tagging, that it is mainly addressed to synaptic terminals. In addition, we observed that CG31030 is essential for fly survival and that this protein co-immunoprecipitates with identified V-ATPase subunits, and in particular ATP6AP2. Using a genetically-encoded pH probe (VMAT-pHluorin) and electrophysiological recordings at the larval neuromuscular junction, we show that *CG31030* knock-down induces a major defect in synaptic vesicle acidification and a decrease in quantal size, which is the amplitude of the postsynaptic response to the release of a single synaptic vesicle. These defects were associated with severe locomotor impairments. Overall, our data indicate that CG31030, which we renamed VhaAC45-related protein (VhaAC45RP), is a specific regulator of neuronal V-ATPase in *Drosophila* that is required for proper synaptic vesicle acidification and neurotransmitter release.

## Significance Statement

In this study, we provide evidence that a previously uncharacterized *Drosophila* protein, CG31030, is necessary for fly survival and expressed specifically in neurons, where it interacts with constitutive and accessory subunits of the V-ATPase. Physiologically, we show that this protein is required for synaptic vesicle acidification and to ensure proper synaptic transmission at the neuromuscular junction. This implies that CG31030, alias VhaAC45RP, is a novel synaptic protein essential to nervous system functioning and neurotransmitter release. This work therefore provides a new step toward a more exhaustive understanding of the regulations of neuronal V-ATPase and their potential repercussions on synaptic transmission and neurologic diseases.

## Introduction

Many cellular processes require a specific electrochemical environment for correct functioning, such as posttranslational modifications of proteins in the Golgi apparatus, lysosomal degradation, endosomal ligand-receptor dissociation, or hormone concentration (for review, see Forgac, 2007). Eukaryotic cells use a highly conserved proton pump, called the vacuolar H^+^-ATPase (V-ATPase), to achieve the adequate level of acidity in different cellular compartments (Saroussi and Nelson, 2009). This large enzymatic complex must be tightly regulated, as it is essential for it to be localized on the right membrane, and to fit the different pH ranges specific to each organelle and cell type.

In neurons, the V-ATPase plays a crucial role at the synapse, being responsible for acidifying synaptic vesicles and thus providing the driving force for neurotransmitter loading (Moriyama et al., 1992). Recently, neuronal V-ATPase has also gained interest in the context of aging and neurodegenerative diseases, as its dysregulation, and resulting impairment of the autophagy-lysosomal pathway, have been linked to several pathologies such as Alzheimer’s and Parkinson’s diseases (Colacurcio and Nixon, 2016; Collins and Forgac, 2020). If the core mechanism of the proton pump is now well understood, the regulations conferring the cell-specific functions of neuronal V-ATPase remain largely unknown, considerably limiting its potential use as a therapeutic target.

The V-ATPase complex is composed of a cytoplasmic domain (V_1_) and a membrane-bound domain (V_0_). The V_1_ domain contains the catalytic unit responsible for ATP hydrolysis. The energy resulting from this reaction powers a rotational molecular motor spanning from V_1_ to V_0_, allowing protons to cross membranes through the port contained in V_0_ (Vasanthakumar and Rubinstein, 2020). The assembly of V_1_ to V_0_ is necessary for the pump to function, and reversible dissociation of the two domains has been shown to occur as a way to regulate V-ATPase activity (Collins and Forgac, 2020). Though the core mechanism stays the same, one V-ATPase can differ from another by its composition. In vertebrates, as well as in *Drosophila*, V_0_ is made of five subunits (*a*, *c*, *c’’*, *d*, and *e*), while V_1_ contains eight subunits (A–H; Allan et al., 2005). Each subunit can have several paralogs encoded by different genes, and each gene can produce several isoforms, allowing many different possible combinations to form the full V-ATPase complex. These differences of composition can also have regulatory effects on the complex, both on its localization and on its functional properties (Vasanthakumar and Rubinstein, 2020).

The V-ATPase can also be regulated by two accessory subunits, ATP6AP1/Ac45 and ATP6AP2/PRR (Jansen and Martens, 2012). Both proteins are found in the nervous tissue but are also required in other organs, and their mutations have been linked to cognitive impairments as well as systemic symptoms like immunodeficiency or hepatopathy (Jansen et al., 2016; Cannata Serio et al., 2018). These two subunits interact directly with the V_0_ domain and are believed to promote assembly of the membrane and soluble regions of the V-ATPase complex (Abbas et al., 2020). In addition, two homologs of ATP6AP1 have been described. The first one, called AC45-like protein (AC45LP), was identified in *Xenopus* where, unlike the ubiquitous AC45/ATP6AP1, it is expressed specifically in kidney and lung (Jansen et al., 2010). The second one, called ATP6AP1-like (ATP6AP1L) or Ac45-related protein (Ac45RP), was recently functionally characterized in mice where it also shows tissue-specificity, its expression being restricted to neurons (Jansen et al., 2021). These studies suggest that the AC45 family is larger than initially expected, and might play an important role in the tissue-specificity of the V-ATPase complex. *Drosophila* possesses identified orthologs of vertebrate ATP6AP1/Ac45 and ATP6AP2/PRR, named VhaAC45 and ATP6AP2, respectively. These proteins also seem to contribute to assembly of the V-ATPase in fly tissues (Schoonderwoert and Martens, 2002a; Guida et al., 2018).

In this study, we examined the localization and function of CG31030, a novel *Drosophila* ortholog of both ATP6AP1/Ac45 and ATP6AP1L/Ac45RP, whose characteristic is to be expressed selectively and ubiquitously in neurons. Whereas a complete deficiency of this protein is lethal, we found that partial *CG31030* knock-down in larval motoneurons impaired synaptic vesicle acidification, reduced quantal size, which is the amplitude of the postsynaptic response to the release of a single synaptic vesicle, and induced severe locomotion defects. We also report that CG31030 from brain tissue co-immunoprecipitated with V-ATPase subunits of the V_0_ domain. Overall, our results indicate that CG31030 is a novel accessory subunit of the neuronal V-ATPase that appears to be involved in the regulation of synaptic activity.

## Materials and Methods

### *Drosophila* culture and strains

Flies were raised on standard agar-cornmeal-yeast medium, at 25°C in a 12/12 h light/dark cycle. *CG31030* RNAi strains and mutants were obtained from the Bloomington *Drosophila* stock center (BDSC) and the Vienna *Drosophila* Resource Center (VDRC). Detailed genotypes and references of these lines are provided in Table 1. To construct the *UAS-CG31030* strain, the *CG31030* cDNA was PCR amplified from the clone RH09162 obtained from the *Drosophila* Genomics Resource Center, using the following primers with added restriction sites: P1-EcoRI (forward) 5′-CCATCCGAATTCAAAATGCAGCTGATTCTCGT and P2-XhoI (reverse) 5′-TGGCTGCTCGAGATCTATTGGGTTATGAGAGA. The 1160-bp PCR fragment was inserted into pUAST (Brand and Perrimon, 1993), verified by sequencing (GATC Biotech) and sent to BestGene, for P-element transformation by random insertion in *w*^1118^ background. A 2d-chromosome insertion of *UAS-CG31030* that yielded strong expression of the transgene was used thereafter.

**Table 1:** *Drosophila* strains used in this study

Name	Genotype	Usage	Sources	References
*w* ^1118^	*w* ^1118^	Wild-type control line	In-house collection	Hazelrigg et al. (1984)
*UAS-CG31030*	w; P*{w*^+^^m^^W.^^hs^=*CG31030*^UAS^*}; +*	Expression of *Drosophila CG31030*	In-house construct	This report
*CG31030* ^V5^	*w; CG31030* ^V5^	Insertion of a V5 tag in *CG31030*	In-house construct	This report
*CG31030* ^RNAi1^	*y*, *sc*, *v*, *sev*^21^; *P{y*^+t7.7^ *v*^+t1.8^*=TRiP.HMC05568}attP40*	*CG31030* knock-down	BDSC no. 64549	Perkins et al. (2015)
*CG31030* ^RNAi2^	*y*, *w*^1118^ ; *P{KK106825}VIE-260B*	*CG31030* knock-down	VDRC no. 107398	Dietzl et al. (2007)
*CG31030* ^RNAi3^	*w*^1118^; *P{GD381}v33095*/TM3	*CG31030* knock-down	VDRC no. 33095	Dietzl et al. (2007)
*CG31030* ^MI107^	*y*, *w*; *Mi{y*^+^^m^^D^^int^^2^*=MIC}CG31030*^MI00107^/*TM3*, *Sb*, *Ser*	MIMIC *CG31030* mutant	BDSC lno. 30620	Nagarkar-Jaiswal et al. (2015)
*Df(3R)Exel6214*	*w*^1118^; *Df(3R)Exel6214*, *P{w*^+mC^=*XP-U}Exel6214*/*TM6B*, *Tb*	Deficiency covering *CG31030* and ∼20 other genes	BDSC no. 7692	Parks et al. (2004)
*elav-Gal4*	*P{w*^+^^m^^W.^^hs^=*GawB}elav*^C155^	Pan-neuronal driver	BDSC no. 458	Lin and Goodman (1994)
*OK371-Gal4*	*w*^1118^; *P{w*^+^^m^^W.^^hs^=*GawB}VGlut*^OK371^	Glutamatergic and motor neuron driver	Gift of Hermann Aberle, Heinrich Heine University, Düsseldorf, Germany	Mahr and Aberle (2006)
*UAS-VMAT-pHluorin*	*w; Vmat* ^UAS.^ ^p^ ^H^ ^luorin^	Expression of *Drosophila* Vmat fused to the pH-sensitive fluorescent marker ecliptic pHluorin	Gift of David Krantz, University of California, Los Angeles	Wu et al. (2013)

### Reverse transcription-coupled PCR and qPCR

Total RNAs were extracted from 20 heads (or 15 thoraces or 15 abdomens) of 8-d-old flies using the QIAzol Lysis reagent (QIAGEN). The Maxima First Strand cDNA Synthesis kit (Thermo Fisher Scientific, K1671) was used with oligo(dT)20 primers to synthesize the cDNAs. Relative quantitative PCR assays were conducted using a LightCycler 480 and the SYBR Green I Master mix (Roche LifeScience), with *Act5C* as internal control for normalization of mRNA levels. All reactions were performed in triplicate. The specificity of amplification products was assessed by melting curve analyses. The following forward and reverse primers, were used: for *CG31030*, 5′-GGCTTCGTTGTAGGCCAACAGA and 5′-CACCAGGTATCCCAAGTTCCAGA; for *Act5C*, 5′-CGTCGACCATGAAGATCAAG and 5′-TTGGAGATCCACATCTGCTG; for *ATP6AP2*, 5′-ACGATCCCTTCAACCTGGC and 5′-CCTGTCAGCTCGTAGGTCT; for *VhaAC45*, 5′-TGTCCCAAGTGGAGTTTGCC and 5′-TCAGGCCATTTTCCTCGAAGG; for *Vha100-1*, 5′-GGCATCCCCATGTTGGACA and 5′-CGGCGTTCTGATTCACCTCC; for *VhaAC39-1*, 5′-GCAGGCTGACTATCTCAACCT and 5′-GCTCCACAGCATGGTTCCTC; for *Vha16-1*, 5′-AAGTCTGGTACCGGTATTGC and 5′-CCATGACCACAGGAATGATG. PCR assays were performed using the PrimeStar Max DNA polymerase (Takara) and the following primers: for *CG31030-RA/RB*, 5′- TGAGGTGTACGACTGCATAGGA and 5′- GAAAGAACTCGATGGCCAAGGT; for *CG31030-RA*, 5′-CAAGGGATACGGTCGATTGAGCATCAC and 5′- CTTACTCCTGTACGGTGAAGGTCAAC; for *CG31030-RB*, 5′- CAAGGGATACGGTCGATTGAGCATCAC and 5′- ATGAACATGGCTGCCCGAGATTGTTTGCT.

### Protein extraction and Western blotting

A total of 30 *Drosophila* heads were homogenized in 300-μl RIPA buffer (Sigma-Aldrich) containing protease inhibitors (Roche Diagnostics, Complete Protease Inhibitor, Cocktail), using bead tubes and a Minilys apparatus (Bertin Technologies). Proteins samples and Western blotting were processed as previously described (Issa et al., 2018). Briefly, the extracted proteins were mixed with LDS sample buffer and reducing agent (Invitrogen, NuPAGE), heated at 70°C for 5 min and separated in 4–12% Novex NuPAGE Bis-Tris precast polyacrylamide gels (Life Technologies) following the manufacturer’s protocol in a MOPS-SDS running buffer. A semi-dry transfer was done onto polyvinylidene difluoride membranes (GE Healthcare Hybond P 0.45 μm) using a Hoefer TE77 apparatus. The mouse monoclonal anti-V5 (Thermo Fisher Scientific, R960-25) was used, diluted at 1:500. Immunolabeled bands were revealed by ECL RevelBlOt Intense (Ozyme, OZYB002-1000) as chemiluminescent HRP substrate and digitally acquired using the ImageQuant TL software (GE Healthcare Life Science).

### CRISPR/Cas9 gene tagging

**Figure 1.**
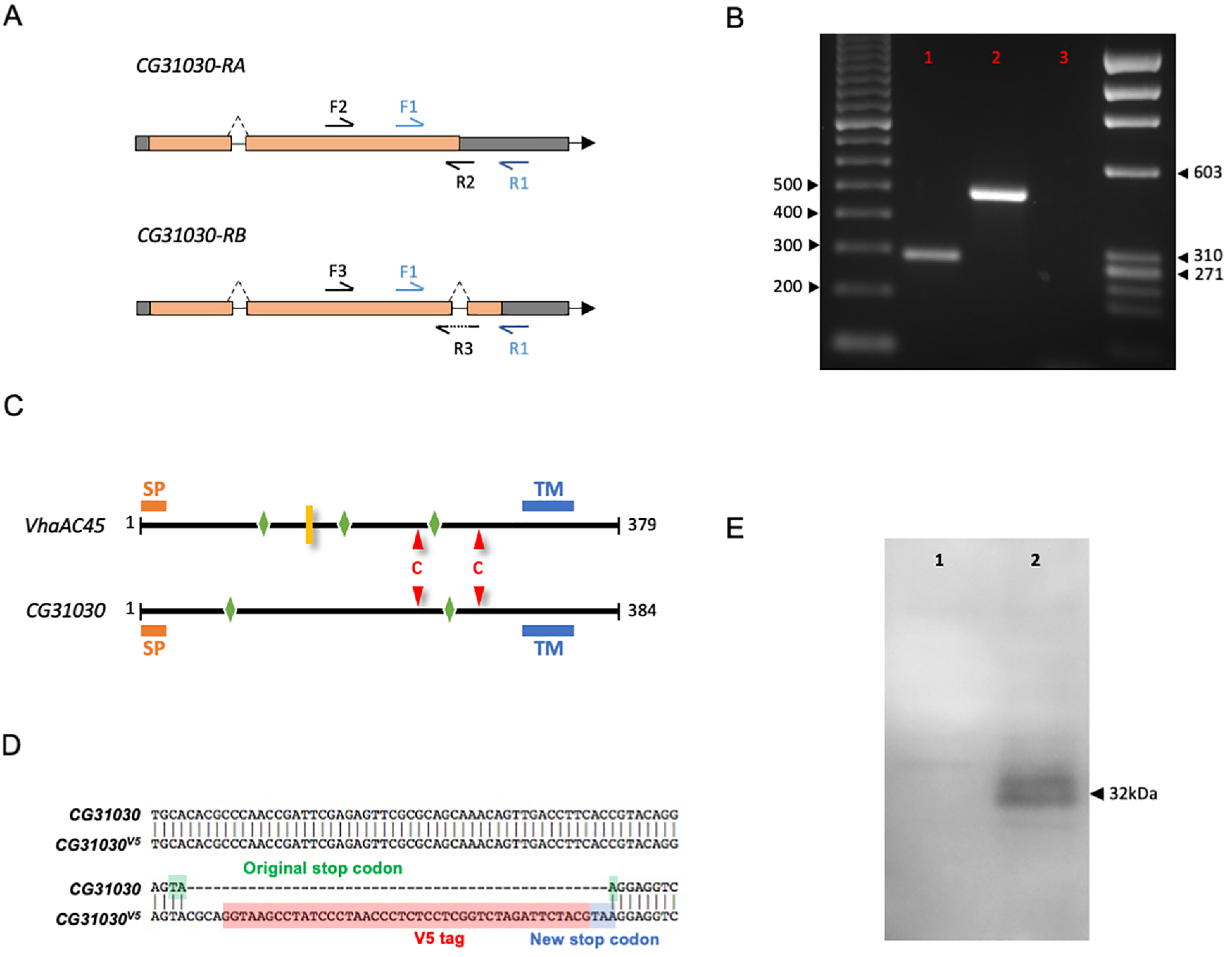
Structure of CG31030 compared with its paralog VhaAC45 and construction of *CG31030*^V5^. ***A***, Localization of the primers used for RT-PCR analysis of the two predicted CG31030 transcripts (*CG31030-RA* and *CG31030-RB*) in *Drosophila* heads. ***B***, Primers detecting both *CG31030-RA* (F1-R1, expected amplicon size 288 bp) and *CG31030-RB* (F1-R1, expected amplicon size 233 bp) only amplify a band corresponding to *CG31030-RA* (lane 1). *CG31030-RA*-specific primers (F2-R2) also amplify a fragment of the expected size (485 bp, lane 2), whereas *CG31030-RB*-specific primers (F3-R3) did not amplify any cDNA fragment (lane 3). ***C***, VhaAC45 and CG31030 both have a signal peptide (SP; orange bars), a transmembrane domain (TM; blue bars) and predicted N-glycosylation sites according to NetNGlyc (http://www.cbs.dtu.dk/services/NetNGlyc/; green diamonds). They also present the pair of cysteine residues characteristic of the AC45 family (red triangles). In addition, VhaAC45 has a predicted furin cleavage site (yellow bar) which is absent in CG31030 according to ProP 1.0 (http://www.cbs.dtu.dk/services/ProP/). ***D***, Construction of the *CG31030*^V5^ mutant strain. A 14-amino acid V5 tag (in red) was fused to the C-terminal end of the CG31030 protein by inserting the V5 coding sequence ended by a new stop codon (in blue) in place of the original stop codon (in green) in the *CG31030* gene using the CRISPR-Cas9 technology. ***E***, A Western blotting probed with anti-V5 antibody detected two protein bands at an apparent molecular weight of ∼32 kDa in head extracts of *CG31030*^V5^ flies (lane 2) and none in the *w*^1118^ control sample (lane 1).

The sequence of a V5 tag was inserted in frame after the coding sequence of the *CG31030* gene, using a homology-directed repair CRISPR-Cas9 method (see scheme in Fig. 1*D*). The following guide RNA sequence: 5′-TTCACCGTACAGGAGTAAGG-3′ was cloned into the BbsI site of *pCFD3-U6:3-gRNA* plasmid (Port et al., 2014; kind gift of Hervé Tricoire, Université de Paris, Paris, France). This plasmid was then injected, at a concentration of 500 ng/μl, with the following single-stranded oligodeoxynucleotide (ssODN) donor repair template: 5′-GTTCGCGCAGCAAACAGTTGACCTTCACCGTACAGGAGTACGCAGGTAAGCCTATCCCTAACCCTCTCCTCGGTCTAGATTCTACGTAAGGAGGTCATAAGTCTCTGATGAACCAATAGATCTCGGGC-3′ (synthetized by Integrated DNA Technology), also at a concentration of 500 ng/μl (the sequence underlined corresponds to the in-frame V5 tag), into *nos-cas9* embryos [genotype y^1^, P(nos-cas9, w+), M(3xP3-RFP.attP)ZH-2A, w*; Port et al., 2014]. An alanine was added before the V5 tag to prevent the creation of a potential tyrosine phosphorylation site. Embryo injections were performed by BestGene. Single F_0_ flies were crossed over the *TM6C*(*Sb*) balancer to establish stable lines. DNA was then extracted from three flies of each of these independent lines, and V5 insertion events were detected by dot blot using a mouse anti-V5 tag monoclonal antibody (Thermo Fisher Scientific, R960-25). Positive strains were outcrossed in a *w*^1118^ background and their genomic DNA was sequenced to check for proper in-frame V5 integration in *CG31030*. One of these equivalent *CG31030*^V5^ mutant line was selected for further studies.

### Immunohistochemistry

Adult brains of 8-d-old females, or third-instar larva CNS, were dissected in *Drosophila* Ringer’s solution or hemolymph-like saline solution (HL3; 70 mm NaCl, 5 mm KCl, 1.5 mm CaCl_2_, 70 mm MgCl_2_, 10 mm NaHCO_3_, 115 mm sucrose, 5 mm trehalose, and 5 mm HEPES, with pH adjusted to 7.6), respectively, and fixed in 4% paraformaldehyde (Thermo Fischer Scientific) for 1 h. After three 20 min washes in PBS plus 0.5% Triton X-100 (PBT), brains were blocked in PBT + 2% bovine serum albumin for 2 h. They were then incubated in primary antibodies diluted in blocking solution for 24 h at 4°C. The primary antibodies used were: mouse monoclonal anti-V5 (Thermo Fisher Scientific, R960-25, 1:200) and rat anti-Cadherin-N (CadN; DSHB, DN-Ex #8, 1:20). Brains were then washed three times, for 20 min each, in PBT before being incubated in secondary antibodies for 2 h. Secondary antibodies used were: Alexa Fluor 488 anti-mouse and Alexa Fluor 555 anti-rat (Fisher Scientific, A11029 and A21434, respectively), all diluted at 1:1000. After two 20-min washes in PBT followed by two 20-min washes in PBS, brains were mounted in Prolong Gold Antifade Mountant (Thermo Fisher Scientific, P36930). Imaged were acquired on a Nikon A1R confocal microscope.

For immunostaining of the larval muscles and neuromuscular junctions, the Alexa Fluor 488 Tyramide SuperBoost kit (Thermo Fisher Scientific, B40912) was used to increase the V5 signal that was otherwise faint in this tissue. The working protocol was as recommended by the manufacturer, with anti-V5 diluted to 1:100, then followed by classical immunostaining, as described above, to co-stain for the nerve terminals with an anti-HRP antibody (Jackson ImmunoResearch, 323-005-021, 1:200) and an anti-Vha100-1 (courtesy of Dr. Peter Robin Hiesinger, 1:2000).

### Longevity assay

Approximately 110 virgin females from each genotype were collected on their hatching day and placed in clean bottles, with no more than 25 flies per bottle. Flies were transferred in new clean bottles, and survivors were counted, every 2 d for 60 d.

### Co-immunoprecipitation coupled to nano liquid chromatography coupled to tandem mass spectrometry (LC-MS/MS) analysis

Approximately 200 heads from 8-d-old *CG31030*^V5^ and *w*^1118^ flies were lysed using glass beads in 500 μl of ice-cold lysis buffer: 50 mm Tris-HCl pH 7.4, 2 mm EDTA, 150 mm NaCl, 0.5% (v/v) IGEPAL CA-630 (Sigma-Aldrich, I3021), 10% (v/v) glycerol, 1 mm PMSF protease inhibitor (Sigma-Aldrich, P-7626), and 1× cOmplete Mini Protease Inhibitor Cocktail (Roche, 11836153001). Samples were left on ice, with occasional gentle agitation, for 30 min before being centrifuged at 12,000 rpm (13,000 × *g*) at 4°C for 10 min to remove insoluble material; 400 μl of the supernatants were then added to 50 μl of Anti-V5-tag mAb-Magnetic Beads (MBL International, M167-11), that had been previously washed as recommended by the manufacturer. Samples were incubated with gentle agitation at 4°C for 4 h. The supernatants were removed using a magnetic rack, and beads were washed three times with 500 μm ice-cold lysis buffer before being resuspended in 10 μl of milliQ water.

Nano LC-MS/MS was performed at the Proteomics facility of the Institut Jacques Monod (Université de Paris). Proteins on beads were digested overnight at 37°C with trypsin (Promega) in 25 mm NH_4_HCO_3_ buffer (0.2 μg trypsin in 20 μl). The resulting peptides were desalted using ZipTip μ-C18 Pipette Tips (Pierce Biotechnology). Eluates were analyzed using either an Orbitrap Fusion or an Orbitrap Q-Exactive Plus, coupled, respectively, to a Nano-LC Proxeon 1200 or a Nano-LC Proxeon 1000, both equipped with an easy spray ion source (Thermo Fisher Scientific).

On the Orbitrap Fusion instrument, peptides were loaded with an online preconcentration method and separated by chromatography using a Pepmap-RSLC C18 column (0.75 × 750 mm, 2 μm, 100 Å) from Thermo Fisher Scientific, equilibrated at 50°C and operated at a flow rate of 300 nl/min. Peptides were eluted by a gradient of solvent A (H_2_O, 0.1% FA) and solvent B (ACN/H_2_O 80/20, 0.1% FA). The column was first equilibrated for 5 min with 95% of A, then B was raised to 28% in 105 min and to 40% in 15 min. Finally, the column was washed with 95% B during 20 min and re-equilibrated at 95% A for 10 min. Peptides masses were analyzed in the Orbitrap cell in full ion scan mode, at a resolution of 120,000, a mass range of *m/z* 350–1550 and an AGC target of 4.10^5^. MS/MS were performed in the top speed 3s mode. Peptides were selected for fragmentation by higher-energy C-trap dissociation (HCD) with a normalized collisional energy of 27% and a dynamic exclusion of 60 s. Fragment masses were measured in an Ion trap in the rapid mode, with and an AGC target of 1.10^4^. Monocharged peptides and unassigned charge states were excluded from the MS/MS acquisition. The maximum ion accumulation times were set to 100 ms for MS and 35 ms for MS/MS acquisitions, respectively.

On the Q-Exactive Plus instrument, peptides were loaded with an online preconcentration method and separated by chromatography using a Pepmap-RSLC C18 column (0.75 × 500 mm, 2 μm, 100 Å) from Thermo Scientific, equilibrated at 50°C and operated at a flow rate of 300 nl/min. Peptides were eluted by a gradient of solvent A (H_2_O, 0.1% FA) and solvent B (100% ACN, 0.1% FA), the column was first equilibrated 5 min with 95% of A, then B was raised to 35% in 93 min and finally, the column was washed with 80% B during 10 min and re-equilibrated at 95% A during 10 min. Peptides masses were analyzed in the Orbitrap cell in full ion scan mode at a resolution of 70,000 with a mass range of *m/z* 375–1500 and an AGC target of 3.10^6^. MS/MS were performed in a Top 20 DDA mode. Peptides were selected for fragmentation by HCD with a normalized collisional energy of 27%, and a dynamic exclusion of 30 s. Fragment masses were measured in the Orbitrap cell at a resolution of 17,500, with an AGC target of 2.10^5^. Monocharged peptides and unassigned charge states were excluded from the MS/MS acquisition. The maximum ion accumulation times were set to 50 ms for MS and 45 ms for MS/MS acquisitions, respectively.

Raw data were processed on Proteome Discoverer 2.4 with the mascot node (Mascot version 2.5.1) against the Swissprot/TrEMBL protein database release 2019_12 for *Drosophila melanogaster*. A maximum of two missed cleavages was authorized. Precursor and fragment mass tolerances were set to, respectively, 7 ppm and 0.5 Da (Orbitrap Fusion) and to 6 ppm and 0.02 Da (Orbitrap Q-exactive Plus). The following posttranslational modifications were included as variable: acetyl (protein N-term), oxidation (M), phosphorylation (STY). Peptide identifications were validated with a 1% FDR (false discovery rate) threshold calculated with the Percolator algorithm. Label-free quantification was done in TOP three abundance calculation mode with pairwise ratio-based calculation and *t* test (background based) hypothesis test. Only proteins identified in at least one group in two independent experiments were kept in the analysis. Missing values were set to the minimum abundance of the experiment.

### VMAT-pHluorin experiments

Third-instar larvae expressing VMAT-pHluorin with or without *CG31030* RNAi in motor neurons using the *OK371-Gal4* driver, were dissected to expose the body wall muscles in Ca^2+^-free HL3 saline solution (70 mm NaCl, 5 mm KCl, 70 mm MgCl_2_, 10 mm NaHCO_3_, 115 mm sucrose, 5 mm trehalose, and 5 mm HEPES, pH 7.6). Two other solutions were used: an acidic Ca^2+^-free HL3 saline (pH 5.5) and a neutral HL3-NH_4_Cl saline (pH 7.6), in which 50 mm NaCl was replaced by 50 mm NH_4_Cl. After dissection, larvae fillets were allowed to settle in Ca^2+^-free HL3 saline for 10 min before being scanned a first time directly in a drop of the solution using a Nikon A1R confocal microscope. Ca^2+^-free HL3 saline was then replaced either by the acidic Ca^2+^-free HL3 saline for low pH-induced quenching experiments, or by the HL3-NH_4_Cl saline for pH gradient collapse experiments. In both cases, larval fillets were rinsed three times with the modified solution, and incubated for 3 min, before being scanned a second time, in a drop of modified solution. Quantification was done on Z-projections (set to maximal intensity) of confocal stacks. Using the Fiji software, a fixed threshold was applied to all images to get rid of the background and select only synaptic areas. Percentage of area over the threshold was used as a measure of the signal intensity and ratios of the values obtained in modified solutions over standard HL3, i.e., the second scan over the first scan, were calculated for quantifications.

### Larval locomotion assays

Larval locomotion was assessed on an in-house made version of the FIMtable system (Risse et al., 2014). Third-instar larvae were collected and briefly rinsed in water to remove traces of food, before being gently placed on the recording table precoated with a thin layer of 1.2% agar gel. Only four larvae of the same genotype were recorded simultaneously, to avoid collisions between animals, for a period of 2 min with a Basler ace acA2040-25gm camera at 12.5 frames/s. Larvae that burrowed themselves into the agar plate or escaped the arena before the end of recording were excluded from the results. Tracking was done using the FIMtrack software, as described in (Risse et al., 2014). The number of peristaltic waves was computed from the variations in larval area. More precisely, the curves of area variation were first smoothed with a Savitzky–Golay filter to get rid of unwanted noise, then the number of waves was defined as the number of peaks on the curve (which was automatically computed by a custom-made Python script). Stride size was then calculated as the distance traveled by the larva divided by the number of peristaltic waves, while stride duration was defined as the recording time (i.e., 120 s) divided by the number of peristaltic waves.

### Electrophysiological recordings and quantal analysis

Third-instar larvae expressing *CG31030* RNAi in motor neurons using the *OK371-Gal4* driver, and appropriate controls, were dissected to expose the body wall muscles, and the brain removed, in Ca^2+^-free HL3 saline solution (70 mm NaCl, 5 mm KCl, 70 mm MgCl_2_, 10 mm NaHCO_3_, 115 mm sucrose, 5 mm trehalose, and 5 mm HEPES, pH 7.6; Stewart et al., 1994; Cattaert and Birman, 2001). Spontaneous miniature EPSPs (mEPSPs) were recorded in the presence of tetrodotoxin (TTX) 10^−6^ m, so that no spike could occur, from the ventral longitudinal abdominal muscle 6 in segment A3.

Quantal analysis was performed following the theoretical background described in (Kuno, 1971) and (Castellucci and Kandel, 1974). Distribution histograms of mEPSP size were built from each muscle fiber recording with a 0.01-mV bin size. These histograms provided an estimate of the mean size of a unitary EPSP, since the peaks represent integer multiples of the unitary size. A theoretical distribution was then computed by convolving a binomial distribution, accounting for quantal content (number of quanta released), and a Gaussian distribution, allowing for variations in size of individual quanta. More precisely, the probability for an EPSP to contain i quanta follows a binomial distribution:

(1) $$\text{P}\left(i\right)=C_{n}^{i}\times p^{i}\times {\left(1-p\right)}^{\left(n-i\right)},$$

in which p is the average probability of release, and n is the total number of releasable quanta. In the frequency distribution of EPSP amplitude, successive peaks represent increasing numbers of quanta. In order to predict how these events are distributed between different bins in a histogram, it is necessary to allow for variations in quantal size. To do this, the largest peaks of the histogram were fitted to a Gaussian curve scaled in width to have a variance proportional to quantal size, and scaled in height so that its area corresponded to the predicted number of events (Del Castillo and Katz, 1954). From the mean (μ) and SD (σ) of the Gaussian curve, the content (*f*) of each bin (*y*) is given by:

(2) $$f\left(y\right)=\frac{1}{\sqrt{2\pi }\sigma }e^{-\frac{1}{2}{\left(\frac{y-\mu }{\sigma }\right)}^{2}}.$$

The SD (σ) of each peak depends on the number of quanta it contains. It was calculated as follows:

(3) $$\text{σ}=i\times \sqrt{{\text{σ}}_{0}},$$

with i the number of quanta in the peak, and ${\text{σ}}_{0}$ the SD of a single quanta. The amplitude of Gaussian distribution for each peak is scaled to the probability P(i) for that peak (see Eq. 1). The complete theoretical distribution, allowing for variance and peak overlap was then obtained by pooling the Gaussians for all peaks. In this way, the theoretical distribution could be superimposed on the histogram for direct comparison with the data.

### Endocytosis and exocytosis measurements

We used fluorescent FM dye labeling to monitor synaptic vesicle cycling at the larval neuromuscular junction (Verstreken et al., 2008). Third-instar larvae expressing mCD8::GFP with or without *CG31030* RNAi in motor neurons under control of the *OK371-Gal4* driver, were dissected in a Ca^2+^-free HL3 saline solution (as described above), and incubated for 5 min in HL3 solution supplemented with 90 mm KCl, 1.5 mm CaCl_2_ and 10 μm fluorescent FM4-64 dye (Invitrogen, T13320) final concentrations. Preparations were then washed with Ca^2+^-free HL3 saline five times, for 2 min each, before being imaged on a Nikon A1R confocal microscope to measure the level of dye loading. Then the fillets were incubated for 2 min in HL3 solution supplemented with 90 mm KCl and 1.5 mm CaCl_2_ to allow the dye to be unloaded from the synapses. Preparations were then washed with Ca^2+^-free HL3 saline five times, for 2 min each, before being scanned once again to measure the level of dye unloading. Images were analyzed using the Fiji software. The fluorescence signal of the dye was measured in the synaptic area restricted to the signal of mCD8::GFP. To obtain the percentage of dye that was unloaded, the ratio of unloaded/loaded fluorescence intensities was calculated.

### Synapse morphology analysis

Immunostaining was performed as described above in third-instar larvae using an antibody against Discs Large (Dlg; DSHB, catalog #4F3 anti-discs large-s, 1:500). Synaptic boutons were then counted manually on the ventral longitudinal abdominal muscle 6 of the segment A3, and their diameter measured in blind on randomized images using the Fiji software.

### Statistical treatment

Statistical analysis was performed with the GraphPad Prism 6 software. The paired Student’s *t* test was used for comparison of two genotypes, while either paired or unpaired ANOVA, with Dunnett’s *post hoc* test for multiple comparisons, were used for three genotypes.

## Results

### *Drosophila CG31030* encodes a single protein structurally similar to VhaAC45

Here, we studied the *Drosophila* gene *CG31030*, identified in FlyBase as a paralog of *CG8029/VhaAC45*, and as an ortholog of vertebrate *ATP6AP1*/*AC45* and *ATP6AP1L/AC45RP* (Thurmond et al., 2019). In FlyBase, *CG31030* is predicted to produce two different protein isoforms, CG31030-PA and CG31030-PB, which differ in the sequence of their C-terminal domain, the last 7 C-terminal amino-acids of CG31030-PA being theoretically replaced by a different segment of 46 amino-acids in CG31030-PB. We first investigated the existence of these two isoforms. Using primers framing the 55 bp difference between the predicted CG*31030-RA* and *CG31030-RB* transcripts (Fig. 1*A*), which encode CG31030-PA and CG31030-PB, respectively, we checked which isoform was present in *Drosophila* heads. Only a fragment with the size expected for *CG31030-RA* was amplified (Fig. 1*B*), suggesting that CG31030-PB is not expressed in this tissue. Consistent with this, PCR experiments using *CG31030-RB*-specific primers did not amplify any cDNA fragment, contrary to experiments using *CG31030-RA*-specific primers (Fig. 1*B*). Moreover, among the numerous cDNA clones matching *CG31030* listed in FlyBase, either fully sequenced or only sequenced at the end (ESTs), most of which complement both isoforms, several were found to be specific to *CG31030-RA*. In contrast, none specifically corresponded to *CG31030-RB*, supporting the hypothesis that among the two predicted protein isoforms, only CG31030-PA is actually expressed in *Drosophila*. Therefore, we referred to CG31030-PA simply as CG31030 in the present study.

CG31030 and VhaAC45 share 69.9% similarity in amino-acid sequences. Like its paralog, *CG31030* is also classified in the V-ATPase family group by the InterPro database (accession Q8IMJ0_DROME; Mitchell et al., 2019). They both have a transmembrane domain, as well as predicted N-glycosylation sites (Fig. 1*C*). They also present a pair of conserved cysteine residues typical of proteins from the AC45 family. Unlike *Drosophila* VhaAC45 and vertebrate ATP6AP1/AC45, CG31030 does not possess a potential furin cleavage site (Fig. 1*C*), similarly to vertebrate ATP6AP1L/AC45RP and *Xenopus* AC45LP (Jansen et al., 2010, 2021).

Using the CRISPR-Cas9 technology, we inserted a small V5 epitope tag in frame at the 3′ end of the *CG31030* gene, thus disrupting the stop codon, to generate the *CG31030*^V5^ mutant line (Fig. 1*D*). A Western blotting on head extracts of *CG31030*^V5^ and control flies was then performed to determine the apparent size of the tagged protein. Two distinct bands, close to each other, were detected specifically in the *CG31030*^V5^ sample, consistent with the presence of glycosylated and unglycosylated forms of the same protein (Fig. 1*E*). While the predicted size of CG31030-V5 is 44 kDa, the observed bands corresponded to a size of ∼32 kDa. This could indicate that the protein is actually cleaved, like its paralog VhaAC45, despite the absence of a predicted cleavage site (Fig. 1*C*). However, it is known that membrane proteins migrate faster on SDS gels, leading to an underestimation of their size (Rath et al., 2009). This could also explain the smaller than expected apparent size of CG31030-V5 on Western blotting.

### CG31030 is specifically expressed in neurons and addressed to synaptic areas

**Figure 2.**
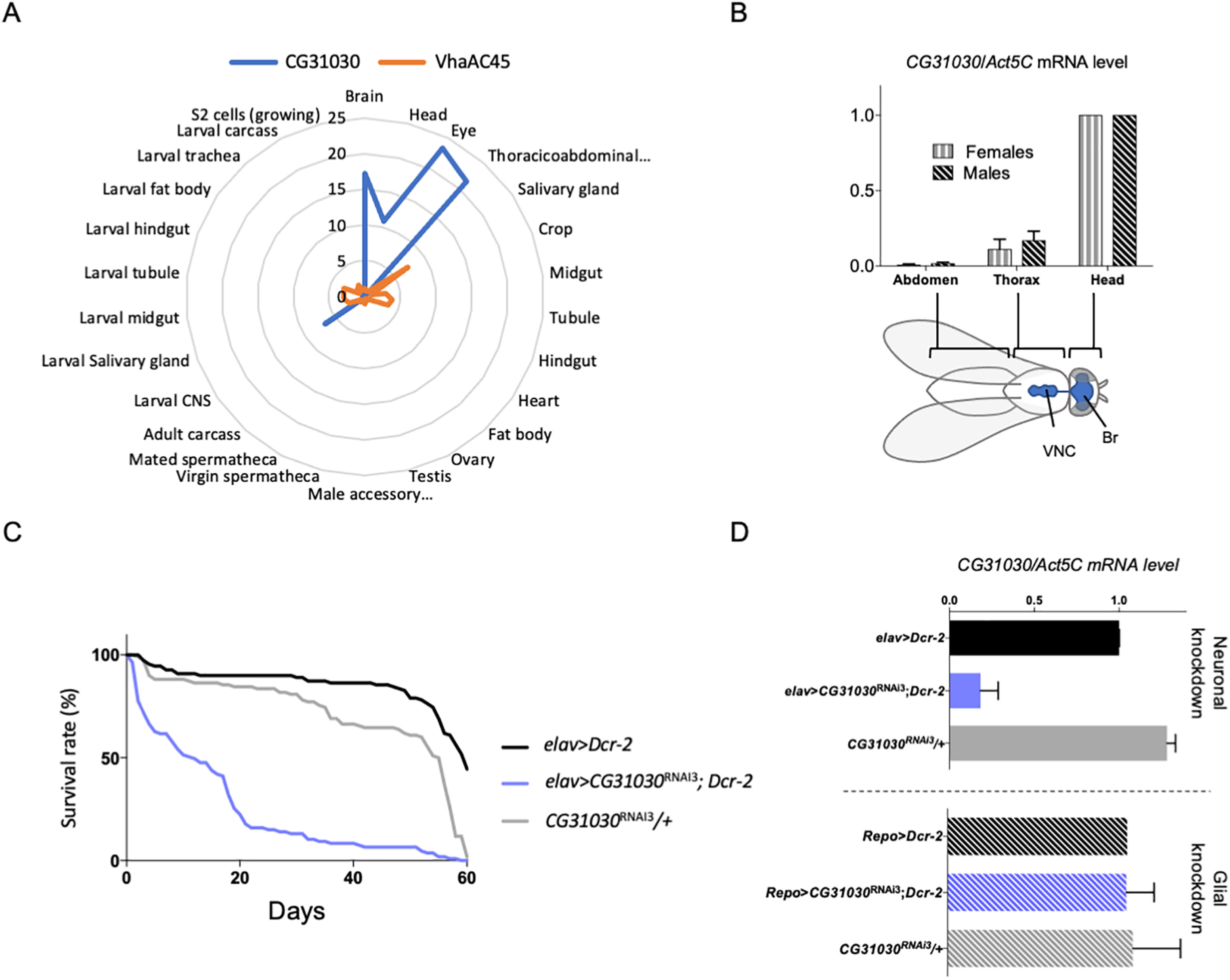
CG31030 is expressed specifically in neurons. ***A***, Diagram representing expression levels of *CG31030* and *VhaAC45* in different tissues relative to the whole fly, according to FlyAtlas data (Chintapalli et al., 2007). CG31030 appears to be markedly enriched in the nervous system of larva and adult fly, whereas in contrast VhaAC45 seems to be uniformly expressed in all tissues in these two stages. ***B***, In both males and females, *CG31030* mRNA abundance follows the localization of the CNS (shown in blue on the fly sketch), with the highest expression in the head. Br, brain; VNC, ventral nerve cord. Results of three independent experiments. ***C***, Knock-down of *CG31030* expression in neurons decreases adult longevity. Pan-neuronal expression of *CG31030*^RNAi3^ and *Dcr-2* with the *elav-Gal4* driver led to a marked shortening of the lifespan of adult flies. Experiment conducted with 105–110 females per genotype. ***D***, Expression of *CG31030*^RNAi3^ with *Dcr-2* in all neurons using *elav-Gal4* decreased *CG31030* mRNA level in head by >80%, while expression of the RNAi construct and Dcr-2 in all adult glial cells with *repo-Gal4* had not effect. Results of three independent experiments. Mean values with SD are reported on the graphs.

According to FlyAtlas (Chintapalli et al., 2007), *VhaAC45* is expressed ubiquitously in *Drosophila* tissues. In contrast and interestingly, *CG31030* seems to be specifically expressed in the nervous system in both larval and adult flies, making it a possible candidate for specific regulation of neuronal V-ATPase (Fig. 2*A*). To confirm this prediction, we checked by RT-qPCR the repartition of *CG31030* transcripts in three parts of the fly body: the head and thorax, which contains the brain and ventral nerve cord (VNC), respectively, and the abdomen, which is relatively poor in nervous tissue. Both females and males showed highest expression in the head, minor expression in the thorax and no detectable expression in the abdomen (Fig. 2*B*). The expression of *CG31030* therefore closely follows the repartition of the nervous system in adult flies.

The single-cell RNA-Seq Scope database (Davie et al., 2018) furthermore indicated that, in *Drosophila*, CG31030 is expressed in all neurons, with few or no expression in glial cells. To verify this, we expressed three different *CG31030* RNAi with either the pan-neuronal driver *elav-Gal4* or the pan-glial driver *repo-Gal4* (see Table 1 for genotypes of the different *Drosophila* lines used in this study). The expression of *CG31030*^RNAi1^ and *CG31030*^RNAi2^ was found to be lethal at embryonic and first larval stages, respectively, while *CG31030*^RNAi3^ produced viable adults with a shortened lifespan (Fig. 2*C*), and obvious locomotor impairments. This difference in phenotypes observed with different RNAi constructs could be attributed to a variation in residual levels of the CG31030 protein. RT-qPCR experiments showed that the pan-neuronal expression of *CG31030*^RNAi3^ together with the RNAi booster *Dicer-2* (*Dcr-2*) was sufficient to decrease by >80% *CG31030* transcripts abundance in extracts from the adult heads (Fig. 2*D*). On the other hand, glial expression of this RNAi construct with *repo-gal4* had no significant effects on *CG31030* transcript level (Fig. 2*D*), confirming that this gene is selectively expressed in neurons. It is interesting to note that both *CG31030*^RNAi1^ and *CG31030*^RNAi2^ also induced lethal phenotypes at various developmental stages when expressed either with a glutamatergic (*VGlut-Gal4*) or a cholinergic (*Cha-Gal4*) neuronal driver (data not shown), in accordance with the RNA-Seq data of the Scope website suggesting a pan-neuronal expression of this gene.

To validate these observations, we placed the available MIMIC allele *CG31030*^MI107^ that contains a stop codon inserted in the middle of the gene (Nagarkar-Jaiswal et al., 2015) over the deficiency *Df(3R)Exel6214* encompassing *CG31030* (Parks et al., 2004). The resulting mutant, likely to be a null, was found to be embryonic lethal, in agreement with the results obtained with two *CG31030* RNAi lines. Remarkably, re-expressing the gene selectively in neurons, using a *UAS-CG31030* construct driven by *elav-Gal4*, was sufficient to rescue this lethality, producing viable and fertile adults with no obvious behavioral defects (Table 2). These results convincingly indicate that *CG31030* is an essential gene whose expression appears to be specifically required in neurons.

**Table 2:** Pan-neuronal expression of *CG31030* rescues the embryonic lethality of CG31030-deficient flies up to the adult stage

Genotype of the progeny flies^1^	Expected percentage^2^	Scored percentage^3^
Heterozygous deficiency without *CG31030* expression	40	39.7
Heterozygous deficiency with pan-neuronal *CG31030* expression	40	53.8
Homozygous deficiency without *CG31030* expression	0	0
Homozygous deficiency with pan-neuronal *CG31030* expression	20	6.4

1 Rescue of embryonic lethality was assayed by crossing *elav-Gal4*; *CG31030*^MI107^/*TM6B*(*Tb*) females with *w*; *UAS-CG31030*/*CyO*; *Df(3R)Exel6214*/*TM6B*(*Tb*) males, and scoring the relative number of CG31030-expressing adult flies homozygous for *CG31030* deficiency (i.e., non-*Tb* and non-*Cy elav-Gal4/+*; *UAS-CG31030/+*; *CG31030*^MI107^/*Df(3R)Exel6214*) in the progeny (highlighted line).

2 Expected percentage of adult progeny flies of each genotype in case of full rescue.

3 Actual percentage obtained in the experiments. 24 rescued adults were recovered out of a total of 373 progeny flies.

**Figure 3.**
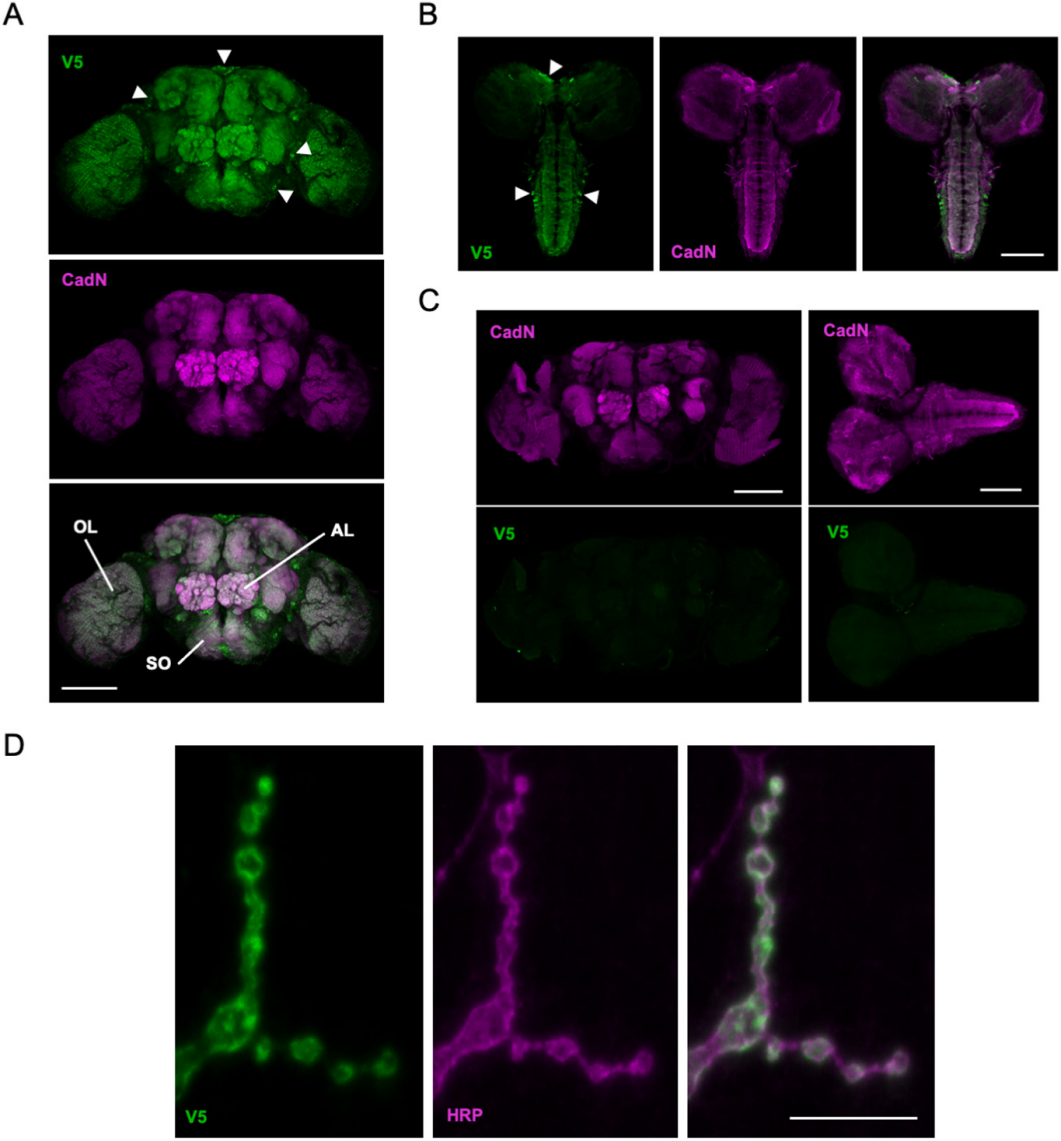
CG31030 is addressed to synaptic areas. ***A***, ***B***, Anti-V5 immunostaining in the *CG31030*^V5^ strain revealed that CG31030 is mainly addressed to synaptic areas in the adult brain (***A***) and larval CNS (***B***), as indicated by its colocalization with the presynaptic marker CadN. Green fluorescence outside synaptic areas reveal that cell bodies are also faintly marked by the V5 antibody, with a few of them showing a bright signal (arrowheads). OL, optic lobe; AL, antennal lobe; SO, subesophageal ganglion. ***C***, CadN and V5 immunostaining of adult brain (left panels) and larval CNS (right panels) from *w*^1118^ control flies. No signal was detected with the anti-V5 antibody in the absence of V5-tagged protein. ***D***, Anti-V5 antibody also labels the neuromuscular junction of *CG31030*^V5^ larvae, where it co-localizes with anti-HRP immunostaining that specifically marks neuronal membranes, confirming CG31030 synaptic localization. Scale bars: 100 μm (***A–C***) and 10 μm (***D***).

**Figure 4.**
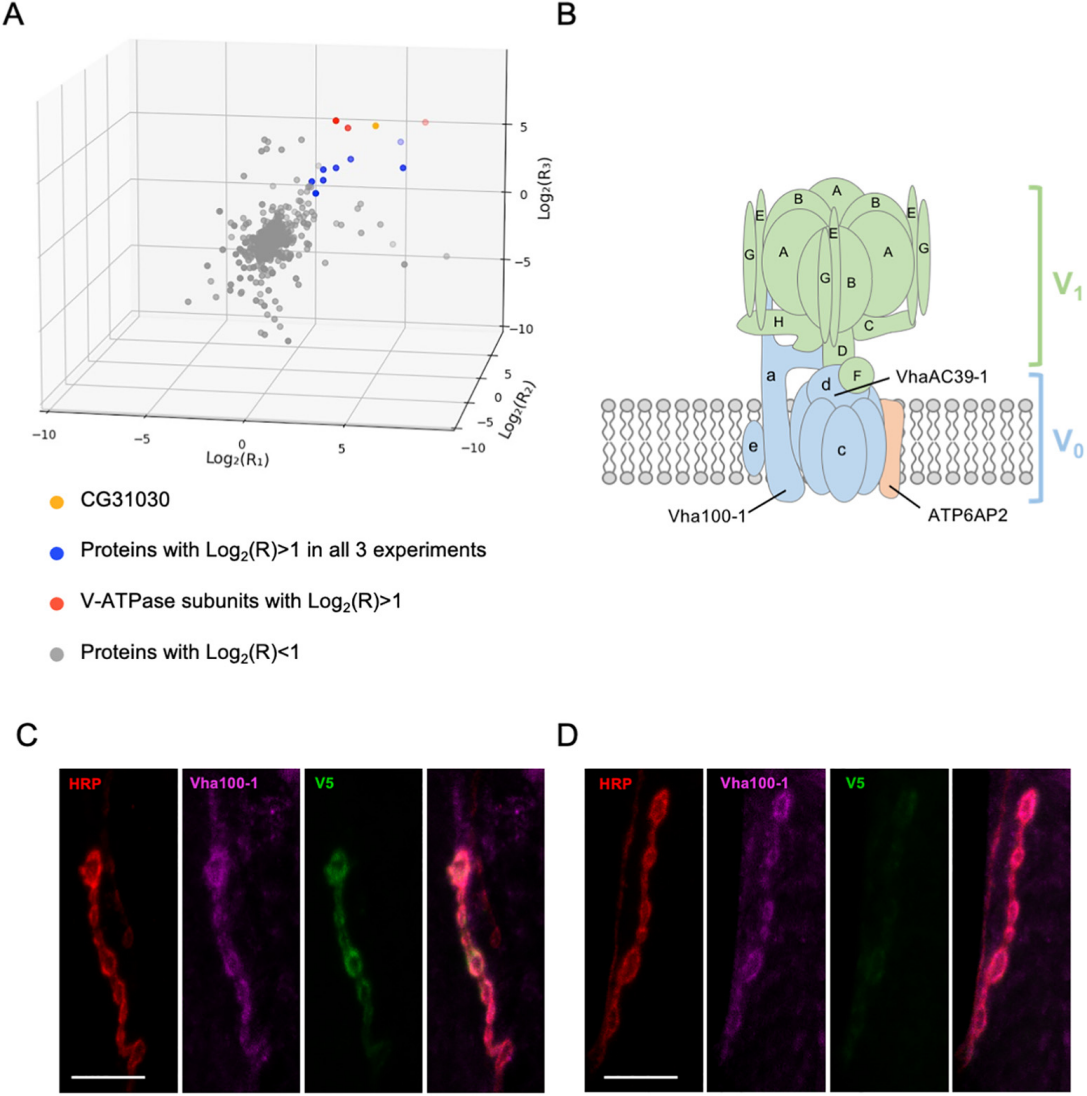
CG31030 co-immunoprecipitates with V-ATPase subunits. ***A***, Scatter plot of proteins identified by nano LC-MS/MS in three independent co-immunoprecipitation experiments using anti-V5 antibodies. R_1_, R_2_ and R_3_ represent the abundance ratio of proteins identified in adult head extracts from *CG31030*^V5^ over *w*^1118^ control in experiments 1, 2 and 3, respectively. Log_2_(R) = 1 corresponds to a 2-fold abundance difference. A total of 12 proteins were found to be at least twice as abundant in *CG31030*^V5^ as in the control in all three experiments (red or blue dots on the graph). One of them is the immunoprecipitation target CG31030 (yellow dot) and three of these proteins belong to the V-ATPase complex (red dots). A list of these 12 proteins with their Log_2_(R) values is provided in Table 3. ***B***, Standard model of the *Drosophila* V-ATPase complex showing structure of the V1 and V0 domains and the predicted localization of the three subunits that co-immunoprecipitated with CG31030. ***C***, Co-localization of CG31030-V5 with Vha100-1 at the larval neuromuscular junction. Larval muscles were labeled with an anti-HRP antibody to mark neuronal membranes. CG31030 has a similar localization to that of Vha100-1, the synaptic isoform of subunit *a* of the V_0_ domain. ***D***, In *w*^1118^ control flies, no specific signal could be detected at the neuromuscular junction with the anti-V5 antibody. Scale bars: 10 μm (***C***, ***D***).

Next, we studied the cellular localization of this protein by performing immunostaining on *CG31030*^V5^ flies with an anti-V5 tag antibody. We observed that the general expression pattern of CG31030 in adult brain (Fig. 3*A*) and larval CNS (Fig. 3*B*) was widespread in the neuropile and quite similar to that of the synaptic marker CadN, consistent with a predominantly synaptic localization. No specific signal was detected in a control *w*^1118^ line that does not contain the V5-tagged protein (Fig. 3*C*). Neuronal cell bodies were also faintly marked with the V5 antibody in the *CG31030*^V5^ line, both in adult and larva, and a few of them displayed a signal intensity comparable to that of the synaptic areas (Fig. 3*A*,*B*, arrowheads). The synapse-containing neuropile areas (like the antennal lobes) were not as sharply defined as with the CadN antibody, suggesting that axons, like most cell bodies, also contains low levels of CG31030. Finally, co-immunostaining of larval body muscles wall with anti-horseradish peroxidase (HRP) antibodies, a marker of *Drosophila* neurons (Jan and Jan, 1982), showed precise co-localization with the V5 signal, indicating that CG31030 is addressed to synaptic boutons at the larval neuromuscular junction (Fig. 3*D*). The lack of V5 immunostaining at the neuromuscular junction of control *w*^1118^ flies is shown below in Figure 4*D*.

**Table 3:** List of the genes encoding proteins that co-immunoprecipitated with CG31030 in *Drosophila* head extracts

Gene symbol^1^	FlyBase gene ID	Log_2_(R_1_)	Log_2_(*R*2)	Log_2_(R_3_)
*ATP6AP2*	FBgn0037671	6.154281057	9.481786693	5.17436482
*Vha100-1*	FBgn0028671	1.499248537	8.012982519	5.43599503
*VhaAC39-1*	FBgn0028665	2.104797507	8.293264658	4.86741949
*CG31030*	FBgn0051030	3.669278876	7.960835791	5,19465574
*Twdlβ*	FBgn0033658	1.642952792	3.068640973	2.35207192
*Ccp84Ag*	FBgn0004777	1.543972087	1.326485589	1.86392045
*CG13627*	FBgn0039217	5.738384187	4.008318188	3.18433108
*mfas*	FBgn0260745	1.596554441	3.368384477	3.03376685
*CG16820*	FBgn0032495	4.77051909	9.784086057	3.60280541
*Cpr64Ab*	FBgn0035511	1.08994458	2.758609792	2.31103817
*CG14752*	FBgn0033307	2.21698759	3.69458382	3.10154816
*CG15615*	FBgn0034159	2.77101281	5.12362053	3.39808341

1 12 proteins were identified with at least twice higher abundance in *CG31030*^V5^ flies compared to the *w*^1118^ control in three independent co-immunoprecipitation experiments with anti-V5 antibodies, followed by mass spectrometry analysis. Among these proteins, three are known to be constitutive or accessory subunits of the V-ATPase complex: ATP6AP2, Vha100-1 and VhaAC39-1.

### CG31030 co-immunoprecipitates with V-ATPase subunits of the V_0_ domain

The CG31030 protein is predicted to be part of the InterPro V-ATPase family, but no experimental information is currently available regarding its potential interactors. To determine whether CG31030 could interact, directly or indirectly, with subunits of the V-ATPase complex, we conducted co-immunoprecipitation experiments using a V5 antibody on proteins extracted from heads of *CG31030*^V5^ mutants and *w*^1118^ control flies, followed by nano LC-MS/MS analysis of the precipitated proteins. Three independent experiments were performed to increase reliability, and in total, 410 proteins were identified in all three experiments. Among those, only 12 proteins had at least a 2-fold abundance difference with the control in all three experiments (Fig. 4*A*; Table 3), one of them being, as expected, the co-immunoprecipitation target CG31030-V5. Remarkably, three of the other proteins were identified as subunits of the V-ATPase complex: Vha100-1, VhaAC39-1, and ATP6AP2 (Fig. 4*B*).

*Vha100-1* and *VhaAC39-1* code for subunits *a* and *d* of the V_0_ domain, respectively (Vasanthakumar and Rubinstein, 2020). The subunit *a* is encoded by five different genes in *Drosophila* and constitutes the proton port of the pump (Collins and Forgac, 2020). Among these five isoforms, Vha100-1 has been shown to be specifically required in neurons and present at the synapse (Hiesinger et al., 2005). Co-immunostaining experiments confirmed that this protein colocalizes with CG31030 at the larval neuromuscular junction (Fig. 4*C*,*D*). Subunit *d* of V_0_ is encoded by two *Drosophila* genes: *VhaAC39-1* and *VhaAC39-2*, and only the first co-immunoprecipitated with CG31030. According to FlyAtlas, VhaAC39-1 is expressed in many tissues and enriched in the brain, while VhaAC39-2 seems to be mostly found in testis and salivary glands. For both V_0_ subunits, CG31030 thus co-precipitated with the likely neuronal isoform. The co-immunoprecipitated V-ATPase subunit which appeared to be the most enriched in the *CG31030*^V5^ sample was, interestingly, the accessory subunit ATP6AP2, suggesting a possible direct interaction between this protein and CG31030 (Table 3). These experiments therefore reinforce the hypothesis that CG31030 directly interacts with the neuronal V-ATPase complex, and more specifically with V_0_ since all detected partners belong, or interact, with this domain.

**Figure 5.**
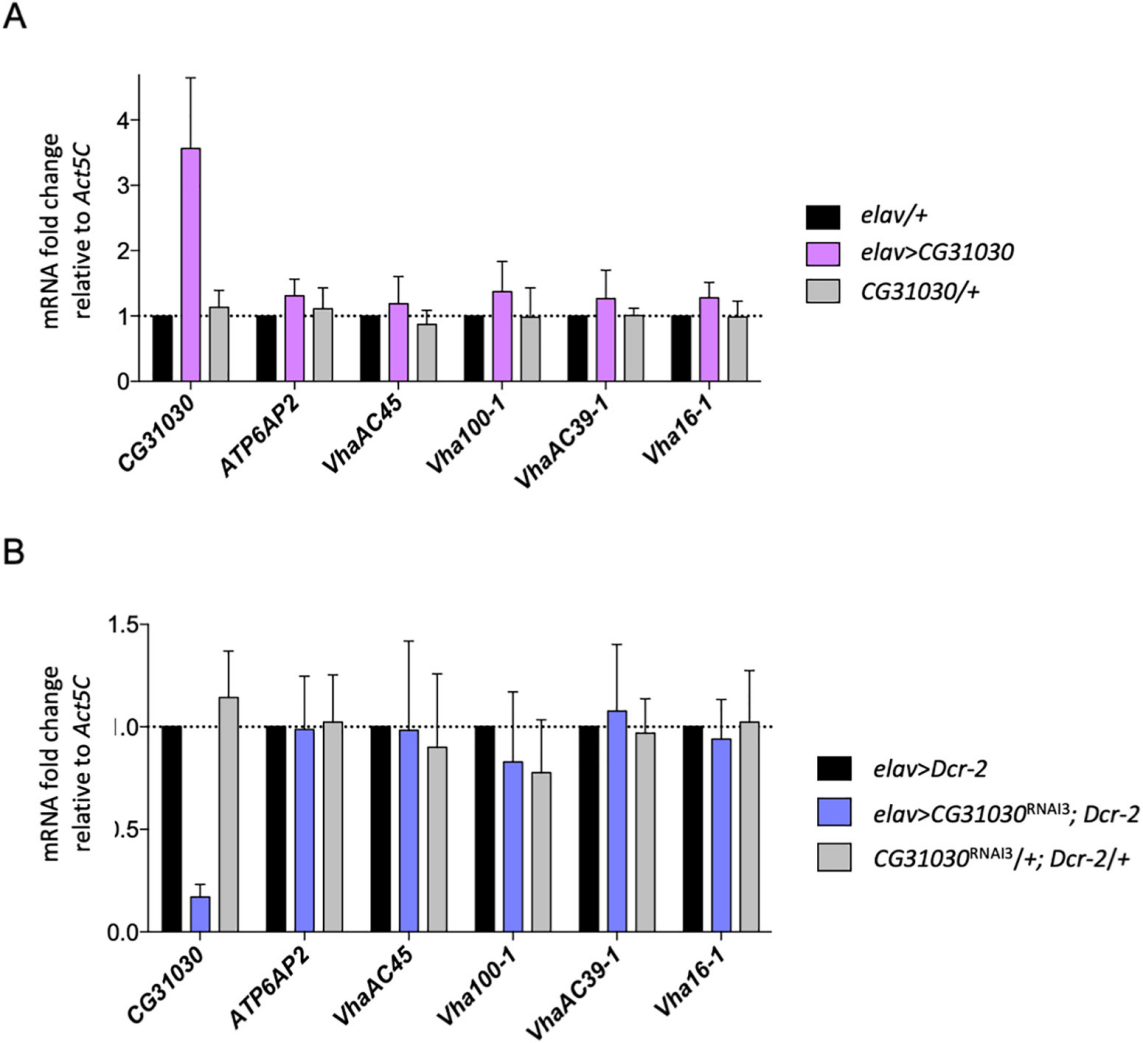
Change in *CG31030* expression does not alter V-ATPase subunit transcript levels. ***A***, ***B***, *CG31030* upregulation (***A***) or knock-down (***B***) *in vivo* did not significantly affect adult head transcript levels of several V-ATPase V_0_ subunits. Results of three independent RT-qPCR experiments. Means and SD are displayed on the graph.

Similarly, the mouse protein ATP6AP1L/AC45RP, a vertebrate homolog of CG31030, has recently been shown to interact selectively with V_0_ domain subunits of the neuronal V-ATPase (Jansen et al., 2021). In addition, these authors showed that strong *in vitro* upregulation (∼7-fold) of this gene resulted in an increased transcript level of some V_0_ subunit, while a moderate one (∼2.6-fold) had no effect except on ATP6AP2. We thus wondered whether *CG31030* upregulation or downregulation could affect V_0_ subunit expression *in vivo*. Using a pan-neuronal driver, we were able to increase *CG31030* transcript levels, but only up to ∼3.5-fold in an *in vivo* wild-type context, suggesting that transcript abundance is regulated posttranscriptionally. This moderate upregulation did not induce any significant changes in transcript levels of V_0_ subunits, but possibly it was not sufficient to see such effects (Fig. 5*A*). Conversely, we performed RT-qPCR experiments on extracts from *Drosophila* heads expressing *CG31030*^RNAi3^ in all neurons compared with controls, and here again, we could not find any significant change in V_0_ subunit expression level in knock-down context (Fig. 5*B*). Notably, *VhaAC45* is expressed at a similar level in *CG31030* knock-down flies and in controls. The homology between CG31030 and VhaAC45 could raise the question of a potential functional redundancy of the two proteins. However, the lethal phenotype and the highly reduced longevity occurring when CG31030^RNAi.1/2^ or CG31030^RNAi.3^ are expressed in all neurons, respectively, suggest that VhaAC45, though still present, is unable to substitute for CG31030.

### CG31030 knock-down impairs synaptic vesicle acidification

**Figure 6.**
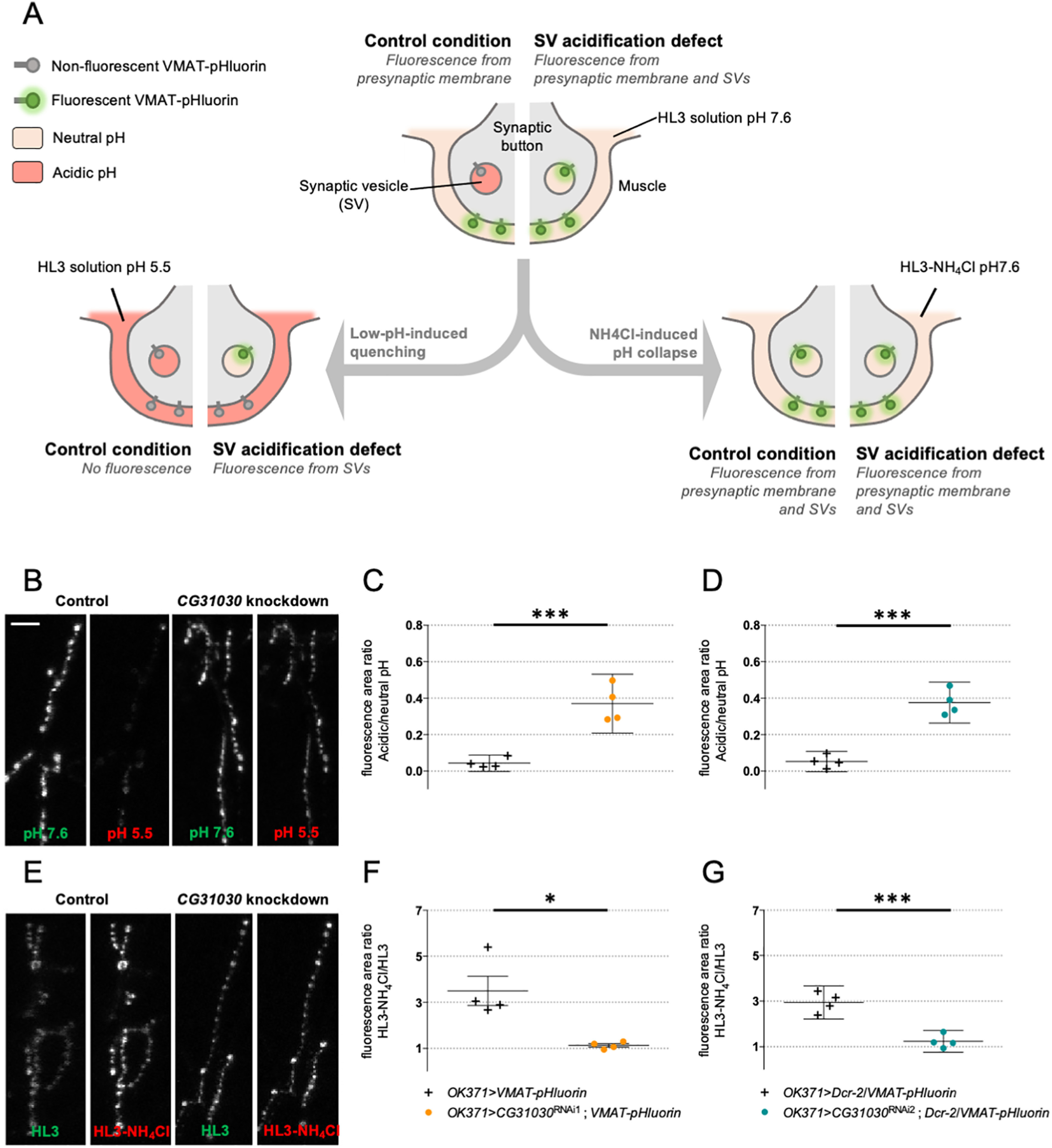
CG31030 knock-down larvae have a synaptic vesicle acidification defect. ***A***, Schematic representation of the protocols used to assess relative acidity levels of synaptic vesicles at the larval neuromuscular junction. Top center diagram, In control conditions, fluorescence can be emitted by VMAT-pHluorin in the presynaptic membrane but not in synaptic vesicles since their lumen is acidified. In case of defective synaptic vesicle acidification, both the presynaptic membrane and synaptic vesicles should emit fluorescence. Left diagram, The fluorescence emitted by VMAT-pHluorin in the presynaptic membrane can be quenched by replacing the extracellular medium with an acidic HL3 solution. This quenching should result in an almost complete extinction of the signal in control flies, in which synaptic vesicles are normally acidified, while a residual signal is expected to be visible in flies having a synaptic vesicle acidification defect. Right diagram, Replacement of 50 mm NaCl by 50 mm NH_4_Cl in the neutral HL3 solution should lead to a collapse of the pH gradients because of the free diffusion of NH3 in membranes, so that fluorescence will be emitted both by the presynaptic membrane and synaptic vesicles both in control and mutant conditions. ***B***, Representative pictures showing the effect of perfusing an acidic HL3 solution on VMAT-pHluorin fluorescence in control and *CG31030* knock-down larvae. ***C***, ***D***, Quantification of the ratio of the fluorescence level at pH 5.5 over the original signal at pH 7.6. Whereas the low pH extinguished fluorescence in control flies, ∼37% of the signal persisted after quenching in *CG31030* knock-down larvae using two different RNAi constructs. ***E***, Representative pictures showing the effect of collapsing the synaptic vesicle pH by perfusing HL3-NH_4_Cl in control and *CG31030* knock-down larvae. ***F***, ***G***, Quantification of the ratio of the signal in HL3-NH_4_Cl over the original signal in HL3 showed that fluorescence increased ∼3-fold in controls while it only rose by 10–20% depending on the RNAi in *CG31030* knock-down larvae. Results of four independent experiments, with three to five larvae analyzed per genotype in each experiment. Unpaired Student’s *t* test; *p <* *0.05, ***p <* *0.001. Mean values with 95% confidence intervals are reported on the graphs. Scale bar: 15 μm (***B***).

Because CG31030 appeared to be mainly localized in synaptic areas, we chose to look at the physiological effect of its disruption at the *Drosophila* larval neuromuscular junction, a model that has contributed to elucidate many essential synaptic processes. At synaptic nerve endings, a prominent role of the V-ATPase is to acidify the lumen of synaptic vesicles, the electrochemical gradient generated providing the driving force to load and concentrate the neurotransmitters. Thus, a malfunction of synaptic V-ATPase should induce a decrease of neurotransmitter concentration inside the vesicles, potentially resulting in an altered synaptic transmission. To test this hypothesis, we co-expressed each of the two strongest *CG31030* RNAi constructs together with VMAT-pHluorin, a pH-sensitive probe targeted to synaptic vesicles (Wu et al., 2013), in larval motoneurons using the glutamatergic driver *OK371-Gal4.* Both RNAi1 and RNAi2 induced a lethal phenotype at pupal stage in these conditions. VMAT-pHluorin is an ecliptic pHluorin that is fluorescent at neutral pH, and gets quenched, by protonation, at acidic pH (Miesenböck et al., 1998). Thus, in control condition, VMAT-pHluorin should not be fluorescent in synaptic vesicles, whose pH is at ∼5.5, but only when externalized on the presynaptic membrane during exocytosis, and so, in contact with the more neutral synaptic cleft milieu. In the case of an acidification defect of synaptic vesicles, the probe could be fluorescent both in synaptic vesicles, where pH would be abnormally high, and on the presynaptic membrane (Fig. 6*A*, central panel).

In order to evaluate the ratio of the internal fluorescence (from synaptic vesicles) over the external fluorescence (from the presynaptic membrane) at the neuromuscular junction of *CG31030* knock-down larvae compared with controls, we first quenched the external signal by replacing the physiological milieu by an identical one with pH adjusted to 5.5 (Fig. 6*A*, left panel). This operation resulted in only the internal signal being conserved. In controls, this meant that all signal was abolished, as expected because the synaptic vesicles were normally acidified (Fig. 6*B*, left panel). In contrast and strikingly, a residual signal was still visible in this acidic milieu in both RNAi1 and RNAi2 knock-down larvae (Fig. 6*B*, right panel). Quantification of the ratio of fluorescence area in acidic milieu over neutral milieu showed that ∼37% of the total signal remained visible in the RNAi larvae after external quenching (Fig. 6*C*,*D*).

To verify that the residual signal seen in knock-down larvae was indeed coming from inside vesicles, the opposite strategy was used: instead of quenching the outside signal, we revealed all the internal one by collapsing the pH gradient of synaptic vesicles (Fig. 6*A*, right panel). To do so, we replaced the physiological milieu by an ammonium solution, as previously described (Poskanzer and Davis, 2004). This solution had the same composition except that 50 mm NaCl were replaced by 50 mm NH_4_Cl. Ammonium and ammonia being in equilibrium (NH_4_^+^ ⇄ NH_3_ + H^+^), uncharged ammonia crosses membranes and binds to protons leading to an alkalization of vesicle lumen pH. This increase of vesicular pH is maintained during NH_4_Cl exposure. In control condition, this gradient collapse should reveal the VMAT-pHluorin probe present in synaptic vesicles and thus highly increase the fluorescent signal. On the contrary, in the case of an acidification defect, the signal should remain fairly stable since synaptic vesicles are already fluorescent. Results were consistent with this hypothesis: while the fluorescence of controls increased ∼3-fold in the pH collapsing ammonium solution, the signal in *CG31030* knock-down larvae hardly rose by 10–20% depending on the RNAi construct (Fig. 6*E–G*). Taken together, these results indicate that *CG31030* knock-down significantly decreases protons concentration in synaptic vesicles of motoneuron terminals.

### CG31030 downregulation decreases larval locomotor performance

**Figure 7.**
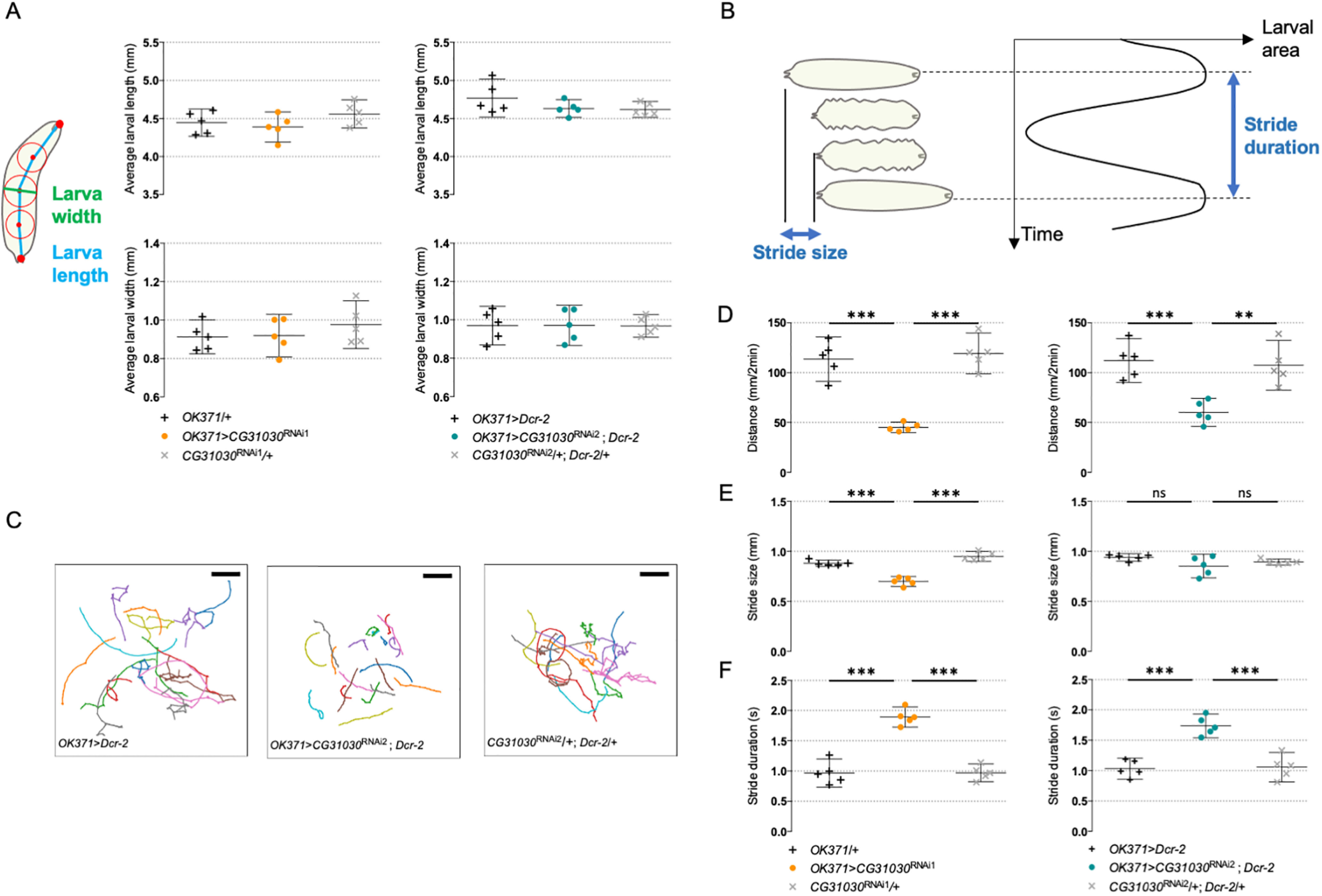
CG31030 downregulation in motoneurons decreases larval locomotor performance. ***A***, CG31030 knock-down in motoneurons did not significantly alter larval size. Larval length is defined as the spine length from head to tail, while larval width is the diameter of the mid-spine circle. Average larval length and width were not significantly different between animals expressing *CG31030* RNAi1 (left panels) and RNAi2 (right panels) in motoneurons and controls. ***B***, Schematic representation of the successive phases of larval locomotion. Stride size is defined as the distance crawled during one peristaltic wave of muscle contraction, while stride duration is the time necessary for the completion of one peristaltic wave. ***C***, Locomotor trails of individual larvae recorded over a period of 2 min. Larvae expressing *CG31030* RNAi in motoneurons show reduced spontaneous movements (middle panel) compared with the driver (left panel) and effector (right panel) controls, respectively. Scale bar: 25 mm. ***D***, Quantification of the traveled distances confirmed that knocking down *CG31030* in motoneurons with two different RNAi induced significant locomotor defects. ***E***, ***F***, Stride size (***E***) appears to be less affected than stride duration (***F***) in the knocked-down larvae. Result of five independent experiments, with three to four larvae analyzed per genotype in each experiment. One-way ANOVA with Dunnett’s *post hoc* test for multiple comparisons; **p <* *0.01, ***p <* *0,001. Mean values with 95% confidence intervals are reported on the graphs.

A reduced pH gradient of synaptic vesicles in larval motoneurons could alter synaptic transmission and, consequently, the larval locomotor behavior. To assess locomotion, we recorded the spontaneous crawling of third-instar larvae expressing *CG31030* RNAi1 or RNAi2 in motoneurons with *OK371-Gal4* on an agar plate with no food source for 2 min periods. We first checked that the average length and width of knock-down larvae were not significantly different from those of control larvae (Fig. 7*A*). Tracking was then performed using the FIMtrack software (Risse et al., 2014), allowing measurement of the total distance traveled and of the stride size, defined as the distance crawled during one peristaltic wave of muscle contraction, and duration (Fig. 7*B*). The knock-down larvae obviously moved less than controls on the plate, as confirmed by their trajectory maps (Fig. 7*C*), traveling a distance only half as long as controls with both RNAi (Fig. 7*D*).

In contrast, the stride size actually showed little or no difference, depending on the RNAi, between knock-down larvae and controls (Fig. 7*E*). Instead, we found that the stride duration, which is the time necessary to accomplish one peristaltic wave, was significantly longer in knock-down animals compared with controls for both RNAi (Fig. 7*F*). These results suggest that the knock-down larvae may need about twice as much time as controls to reach the required level of muscular contraction to accomplish one stride. This could be a compensatory mechanism to adapt to a lower amount of neurotransmitter released in response to motor nerve stimulation, as would be expected if the synaptic vesicles were less filled in *CG31030* knock-down context.

### Quantal size is reduced in *CG31030* knock-down larvae

**Figure 8.**
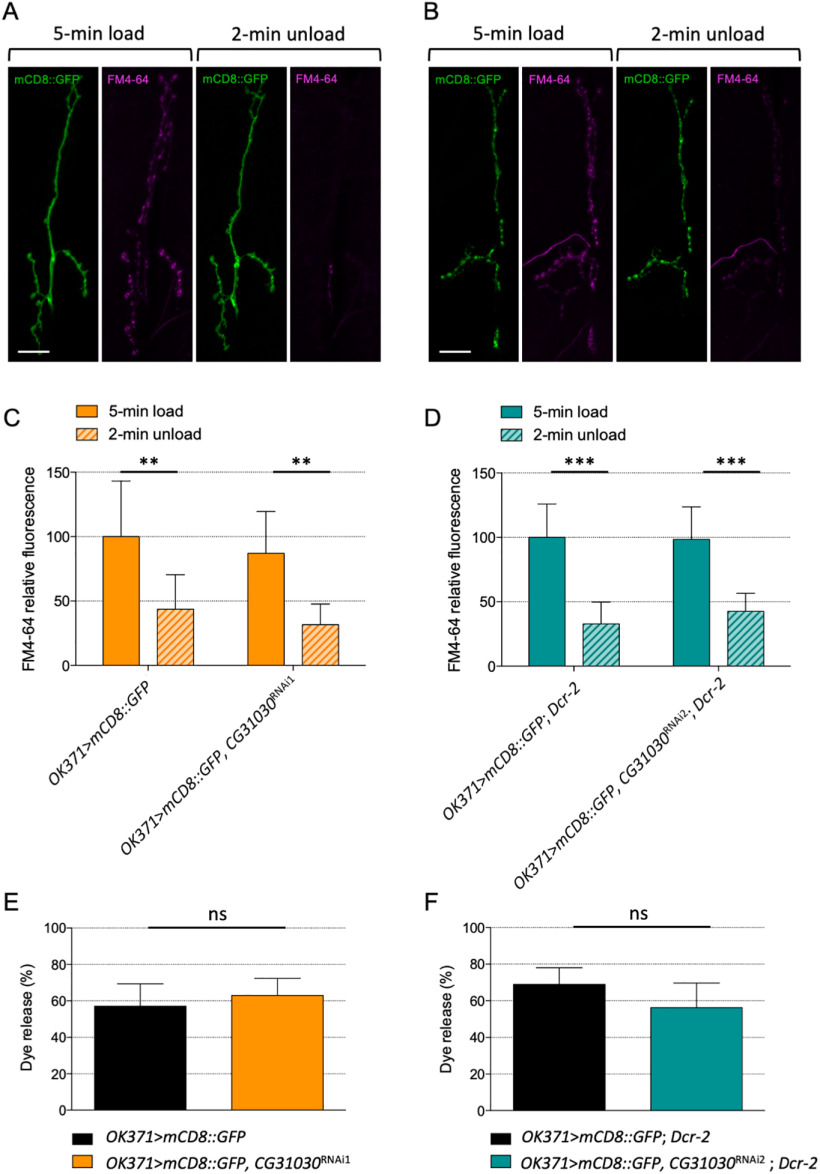
Lack of effect of *CG31030* knock-down on the synaptic vesicle cycle at the larval neuromuscular junction. ***A***, ***B***, Representative images of mCD8::GFP-labeled neuromuscular junctions of a control (***A***) and a knock-down (***B***) larva after loading and unloading of the fluorescent FM4-64 dye. ***C***, ***D***, Nerve terminals from larvae expressing *CG31030* RNAi1 (***C***) or RNAi2 (***D***) were able to both load and unload the fluorescent FM4-64 dye as efficiently as controls. No significant difference was observed between controls and knock-down animals during the loading and the unloading phases. Fluorescence was normalized relative to the level in control larva synapses after 5-min loading. ***E***, ***F***, The percentage of previously loaded FM4-64 dye released during the unloading phase was similar for control and knock-down larvae when using either RNAi1 (***E***) or RNAi2 (***F***); **p < 0.01, ***p 0.001, ns: not significant.

The locomotor deficit of *CG31030* knock-down larvae could originate from an impairment of synaptic transmission at the neuromuscular junction or a defect in the central control of motor behavior, or both. Fluorescent FM-dye loading/unloading experiments can be used to monitor potential defects in endocytosis or exocytosis of synaptic vesicles at the larval neuromuscular junction (Verstreken et al., 2008). We therefore assessed whether the synaptic vesicle cycle induced by KCl stimulation was affected by *CG31030* downregulation, as this could well have an impact on neurotransmission and locomotion. Representative images of neuromuscular junctions from control and knock-down larvae after FM-dye loading and unloading are shown in Figure 8*A*,*B*, respectively. The larvae expressing *CG31030* RNAi1 or RNAi2 in motoneurons, were apparently able to load and unload the dye in synaptic junctions similarly as controls (Fig. 8*C*,*D*). The percentage of previously loaded FM4-64 dye released during the unloading phase was also comparable for control and knock-down larvae (Fig. 8*E*,*F*). This result indicates that the synaptic vesicle cycle, and in particular the exocytosis machinery, were apparently not impacted by *CG31030* inactivation.

**Figure 9.**
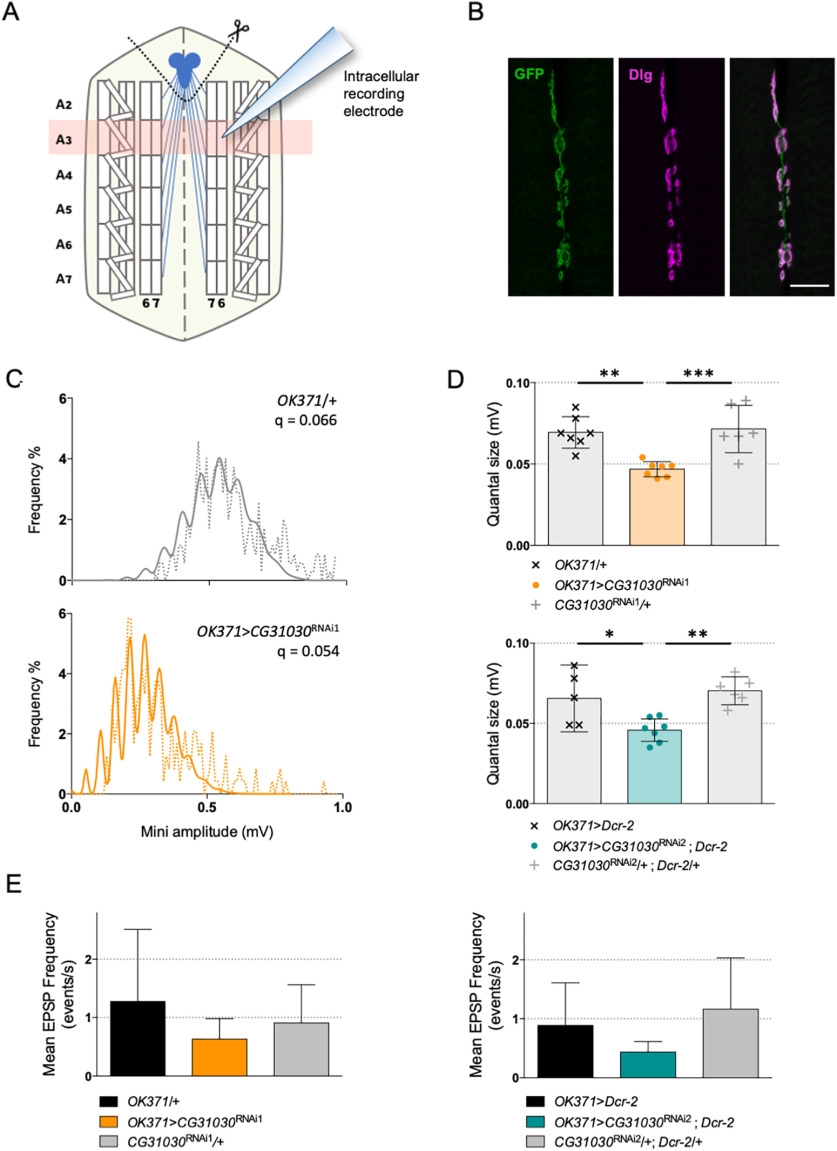
Synaptic quanta size is reduced in *CG31030* knock-down larvae. ***A***, Schematic representation of a dissected larval fillet. Spontaneous miniature EPSPs (mEPSPs) were recorded intracellularly from the ventral longitudinal abdominal muscle 6 in segment A3. ***B***, Expression of membrane-associated mCD8::GFP with the glutamatergic driver *OK371-Gal4* strongly labels the presynaptic nerve endings at the neuromuscular junction of muscles 6–7 in segment A3. The scaffolding protein Dlg was used as a postsynaptic marker. Scale bar: 20 μm. ***C***, Representative distributions of spontaneous mEPSPs recorded in a control larva (top panel, in gray) and a *CG31030* RNAi knock-down larva (bottom panel, in orange). The dotted lines represent actually recorded amplitudes and the plain lines the computed theoretical distributions. Genotypes and quantal size (q) are indicated on each graph. ***D***, Quantal analysis of recorded events showed that knock-down larvae have a significantly reduced synaptic quanta size compared with controls, both with *CG31030* RNAi1 (top panel, in orange) or RNAi2 (bottom panel, in blue). ***E***, Although both RNAi1 (left panel) and RNAi2 (right panel) larvae had a lower mean EPSP frequency than controls, this difference was not statistically significant. Six to seven cells recorded per genotype. One-way ANOVA with Dunnett’s *post hoc* test for multiple comparisons; *p <* *0.05, **p <* *0.01, ***p <* *0.001. Mean values with 95% confidence intervals are reported on the graphs.

To determine whether neurotransmission was otherwise affected by *CG31030* knock-down, we conducted electrophysiological recordings to measure the quantal size, which is the postsynaptic response to the release of one synaptic vesicle. We expressed *CG31030* RNAi1 or RNAi2 in motoneurons of third-instar larvae with the *OK371-Gal4* driver, and recorded spontaneous mEPSPs intracellularly from ventral longitudinal abdominal muscle 6 of segment A3 (Fig. 9*A*). These muscles are innervated by synaptic boutons that are clearly marked by *OK371-Gal4*, as shown by the co-localization of membrane-associated GFP and the postsynaptic marker Dlg in *OK371*>*mCD8::GFP* flies (Fig. 9*B*). Representative amplitude distributions of mEPSPs in a control and RNAi1 larvae are shown in Figure 9*C*. Quantal analysis of recorded events confirmed that both RNAi1 and RNAi2 knock-down larvae have a significantly reduced quantal size compared with controls (Fig. 9*D*), suggesting a decrease in glutamate vesicular uptake, and potentially linking vesicle acidification defect and locomotor impairment. The V-ATPase dysfunction in synapses of *CG31030* knock-down larvae could apparently lead to incomplete loading of vesicles, thus decreasing the amount of neurotransmitter released per unit of time during a peristaltic wave, and potentially slowing down larval locomotion. Although the mean frequency of mEPSPs of both RNAi1 and RNAi2 larvae appeared lower than controls, this effect was not statistically significant (Fig. 9*E*). This may suggest that *CG31030* knock-down did not significantly increase the number of unacidified vesicles empty of neurotransmitter.

**Figure 10.**
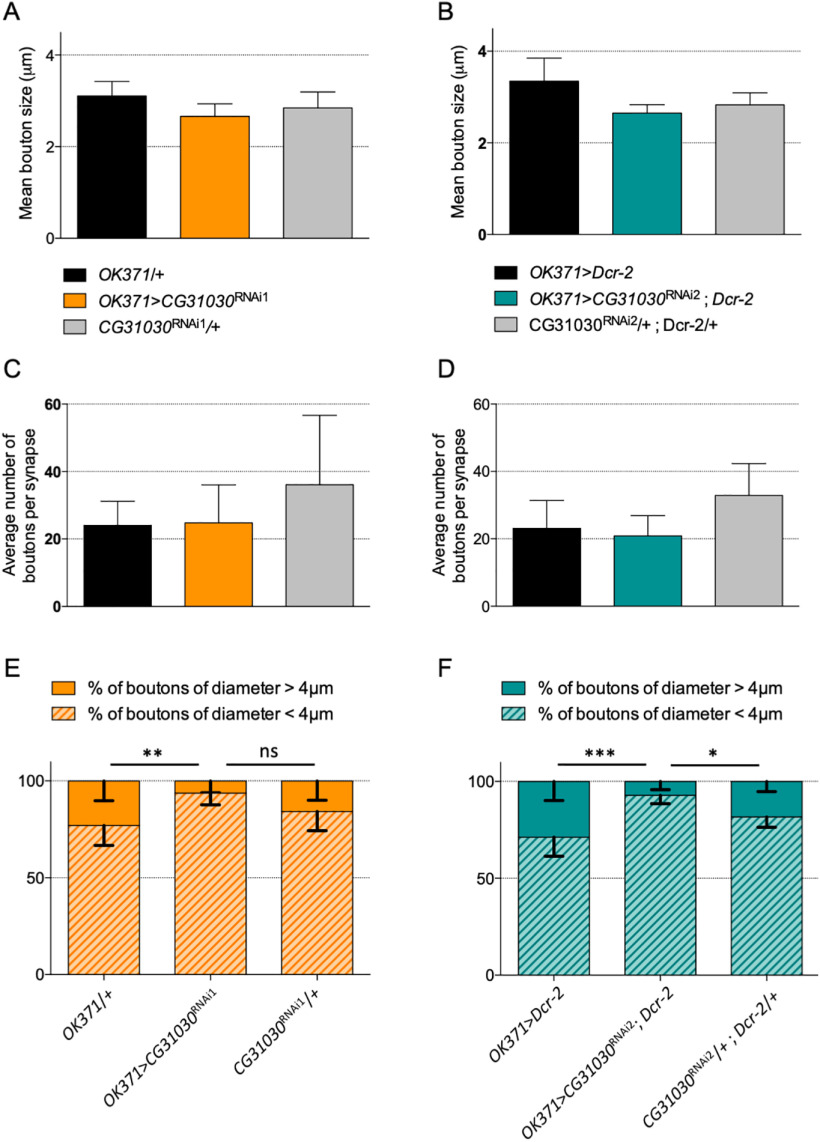
Effect of *CG31030* knock-down on synapse morphology at the larval neuromuscular junction. ***A–D***, No significant difference was observed in the mean synaptic bouton diameter (***A***, ***B***) or the average number of boutons per synapse (***C***, ***D***) between larvae expressing *CG31030* RNAi1 (***A***, ***C***) or RNAi2 (***B***, ***D***) in motoneurons and the controls. ***E***, ***F***, Knock-down larvae showed a smaller percentage of boutons with a diameter larger than 4 μm for both RNAi1 [***E***; but not significant (ns) compared with the effector control] and RNAi2 (***F***), *n* = 6–8 synapses per genotype, two-way ANOVA with Dunnett’s correction for multiple comparisons; *p < 0.05, **p < 0.01, ***p < 0.001, ns: not significant.

Finally, we also looked at the morphology of the neuromuscular junctions in *CG31030* knock-down and control animals by measuring the number and diameter of synaptic boutons innervating the ventral longitudinal abdominal muscle 6 in the segment A3. The overall number and mean size of boutons were not significantly affected between the different genotypes (Fig. 10*A–D*). However, we observed that knock-down larvae exhibited a reduced number of boutons with a diameter superior to 4 μm for both RNAi1 and RNAi2 (Fig. 10*E*,*F*). The fact that these large boutons only represented a small fraction of the total explains why the average number or mean diameter of boutons were not altered in knock-down larvae. It is therefore unlikely that this size variation could result, by itself, in the significant decrease in quantal size and strong locomotor impairment induced by *CG31030* downregulation. Neuronal activity is known to influence synaptic growth at the neuromuscular junction (Menon et al., 2013), so the lower number of larger boutons in knock-down animals could be a consequence of the impaired synaptic transmission resulting from the quantal size decrease. Alternatively, the lower number of high diameter boutons could also be a modest contributor to the observed quantal size decrease, as larger boutons have been shown to harbor bigger synaptic vesicles associated with higher quantal content (Karunanithi et al., 2002).

## Discussion

In this study, we investigated the hypothesis that the previously uncharacterized *Drosophila* protein CG31030 is a specific regulator of the neuronal V-ATPase. At variance with its broadly expressed paralog VhaAC45, we have shown that CG31030 is found mainly, if not only, in neurons. We also provide evidence that CG31030 interacts with two constitutive subunits and one accessory subunit of the V-ATPase, the constitutive ones being also enriched in neurons, and that it is required to have properly acidified synaptic vesicles. This implies that CG31030 is an essential protein for nervous system functioning in *Drosophila*.

### CG31030 is an essential synaptic protein

In yeast, all V-ATPase subunits are coded by a single gene, with the exception of the V_0_ subunit *a*. The knock-out of any of the single-gene subunits all present a similar phenotype: the inability to survive in a neutral pH environment (Nelson, 2003). For subunit *a*, the same phenotype was only achieved in a double-mutant of both isoforms (Manolson et al., 1994). In multicellular organisms, mutations of V-ATPase subunits, or accessory proteins, also often lead to a lethal phenotype at various developmental stages, whether in mice (Inoue et al., 1999; Sun-Wada et al., 2000; Schoonderwoert and Martens, 2002b), *Caenorhabditis elegans* (Lee et al., 2010), or flies (Davies et al., 1996; Allan et al., 2005). Similarly, we found a *CG31030* null mutant to be embryonic lethal, and, interestingly, this lethality could be rescued to the adult stage by re-expressing *CG31030* in all neurons, showing that the protein is specifically required in this cell type for fly survival. The percentage of adult survivors was about three times less than what would be expected in case of a full rescue (Table 2) and the missing two-thirds likely died at an early developmental stage as no larval lethality was observed. This could be explained by the fact that CG31030 protein re-expression in rescued knock-out flies first required the expression of Gal4 under regulation of the *elav* promoter, which starts to express rather late in embryos, followed by activation of the UAS sequence upstream the *CG31030* insert. So, it is possible that part of the mutant embryos did not survive the delay inherent to this ectopic expression process.

We observed that *CG31030* transcripts follows the repartition of the nervous system, with the highest relative abundance in the head. Moreover, pan-neuronal expression of *CG31030*^RNAi3^, the RNAi construct with the weakest effect on fly survival, was sufficient to decrease by >80% its level in the brain of adult escapers. These results indicate that CG31030 expression is mainly neuronal, if not even entirely restricted to neurons. In addition, CG31030 cellular localization shows similarity with a synaptic pattern. Cell bodies were also marked, although generally less strongly. Synaptic protein complexes can be assembled in the cell bodies before being transported in axons, and it is the case for the soluble and membrane-bound domains of synaptic V-ATPase, as was shown in *Torpedo* (Morel et al., 1998). It is difficult to decide whether the signal coming from cell bodies corresponds to a functional site for CG31030 or rather to newly produced proteins that have not yet been targeted to synapses. However, the prominent signal in synaptic areas led us to assume that CG31030 plays an important role in the synaptic process.

### CG31030 is required for proper synaptic vesicle acidification

In presynaptic terminals, the most abundant organelles are synaptic vesicles, which require acidification to provide the driving force for neurotransmitter loading. This acidification is ensured by the V-ATPase pump, which generates an electrochemical gradient by importing protons into the vesicle lumen, thus creating both a membrane potential (ΔΨ, inside positive voltage) and a pH gradient (ΔpH, acidic lumen). Our experiments showed that the knock-down of *CG31030* at the glutamatergic larval neuromuscular junction increased the internal pH of synaptic vesicles, and so decreased the ΔpH component of the electrochemical gradient generated by the V-ATPase. This result is however by itself insufficient to conclude to a dysfunction of the proton pump, since other players have been shown to influence synaptic vesicle pH gradient downstream V-ATPase activity. For example, cation/H^+^ exchangers, found on synaptic vesicles, can decrease ΔpH while increasing ΔΨ by exchanging cations, like Na^+^ or K^+^, against luminal H^+^ (Takamori, 2016). This activity can be upregulated by increased intracellular concentration of these cations (Huang and Trussell, 2014). Glutamate being negatively charged, its transport across vesicular membrane is predominantly driven by ΔΨ (Maycox et al., 1988; Tabb et al., 1992). As a consequence, the increased membrane potential resulting from upregulation of cation/H+ exchangers actually facilitates glutamate uptake (Goh et al., 2011; Huang and Trussell, 2014). We measured synaptic quanta size by quantal analysis of mEPSPs of *CG31030* knock-down larvae and found that it was decreased, contrary to what would be expected in the case of an upregulation of cation/H^+^ exchanger activity. This apparent decrease of vesicular glutamate concentration was corroborated by its phenotypical consequence, namely the locomotion defect exhibited by knock-down larvae. This suggests that the observed diminution of ΔpH is correlated with a diminution of ΔΨ, thus pointing toward a V-ATPase malfunction. This hypothesis is further supported, physiologically, by the lack of defect in the endocytosis/exocytosis cycling of synaptic vesicles in knock-down larvae, making it unlikely that such a defect could be at the origin of the locomotor impairment, and, at the molecular level, by the co-immunoprecipitation of CG31030 with three V-ATPase subunits of the V_0_ domain (Vha100-1, VhaAC39-1, and ATP6AP2), all of them being associated with the neuronal V-ATPase. Altogether, these results strongly suggest that CG31030 is a neuronal protein necessary for V-ATPase function in synaptic nerve endings.

Some V-ATPase subunits isoforms in other species have been shown to target specific subcellular compartments, like the *Torpedo* V_0_ a1 isoform which is found in synaptic V-ATPase and not in neuronal cell bodies (Morel et al., 2003). Similarly, CG31030 could be specifically involved in the regulation of synaptic V-ATPase, or, alternatively, globally act on all neuronal V-ATPase. Specificity level could even be pushed further, since synaptic vesicles are not the only organelles requiring acidification that are localized in synapses. Thus, a synaptic V-ATPase regulator could be devoted to synaptic vesicles as it could be affecting more generally all synaptic V-ATPase complexes, indifferently of their membrane localization. The answer to such questions could help better understand regulations of synaptic transmission, since synaptic V-ATPase activity is one of the presynaptic modulators of quantal response (Takamori, 2016; Gowrisankaran and Milosevic, 2020).

### CG31030 interacts with ATP6AP2 and may regulate V-ATPase domain dynamics

While accessory subunits directly interact with V-ATPase domains, other stimulus can indirectly affect activity of the complex, like glucose concentration or serotonin (Sautin et al., 2005; Voss et al., 2007). Co-precipitation of CG31030 with V-ATPase subunits does not necessarily imply a direct interaction, but suggests a minima an indirect association with the complex, possibly in a non-transient manner. The impairment in V-ATPase activity induced by CG31030 downregulation rules out the hypothesis of an inhibitory action of this protein on the proton pump. Nevertheless, the precise requirement of CG31030 for activity of the V-ATPase complex still remains unknown to date. Lethality of the mutant could indicate an essential role of CG31030 in V-ATPase function. However, some V-ATPase regulators and accessory subunits have been found in other signaling pathways, like ATP6AP2 in the renin-angiotensin system (Nguyen, 2010), so we cannot exclude that lethality could be because of CG31030 playing a part in other vital neuronal functions. Signal in axons could also point to a role in targeting the complex to the synapses. The two domains of the V-ATPase, V_0_ and V_1_, are believed to be assembled in cell bodies before being separately transported to synaptic area, V_0_ by fast axonal anterograde transport, most likely directly on new synaptic vesicles, and V_1_ by slow axonal transport like other cytoplasmic synaptic proteins (Morel et al., 1998). CG31030 could be involved in the transport of one of the two domains, and effects of CG31030 knock-down could then result from a decreased synaptic abundance of the affected V-ATPase domain. However, it is believed that only one copy of the V-ATPase complex is sufficient to properly acidify one synaptic vesicle (Takamori, 2016), a consequence of this being that neurotransmitter loading would be an all-or-none process. Consistent with this, mutation of the *Drosophila* synaptic V_0_ subunit Vha100-1 does not seem to impact quantal size but rather mEPSP frequency, potentially reflecting the presence of an increased number of empty synaptic vesicles (Hiesinger et al., 2005). Similarly, a reduction in the frequency of spontaneous quantal events with no change in quantal size was observed when the *Drosophila* vesicular glutamatergic transporter (VGlut) was downregulated, also suggesting that a single copy of VGlut is sufficient for proper loading of a vesicle (Daniels et al., 2006). The fact that CG31030 knock-down changes the quantal size but not significantly mEPSP frequency seems to point toward a role of this accessory protein in V-ATPase efficiency, rather than in a process affecting the abundance of the complex such as synaptic targeting.

In this respect, it is interesting to note that the V-ATPase protein that more consistently co-immunoprecipitated with CG31030 in our experiments is the accessory subunit ATP6AP2, the fly homolog of human ATP6AP2/PRR. Interestingly, the vertebrate homolog of CG31030, ATP6AP1/Ac45, has also been shown to interact with ATP6AP2, and the complex they form has been proposed to enable the assembly and disassembly of the catalytic and membrane domains of the V-ATPase in the mammalian brain (Rujano et al., 2017; Abbas et al., 2020). This suggests that CG31030 could similarly play a role in the regulation of these dynamic processes. Indeed, it was shown that the dissociation of the two V-ATPase domains on synaptic vesicles is a necessary step before exocytosis (Bodzęta et al., 2017). When new vesicles are formed through endocytosis, the two domains then reassemble to start re-acidifying the vesicular lumen, allowing neurotransmitter uptake. Thus, during the synaptic vesicles recycling cycle, V_0_ and V_1_ dynamically alternate between assembled and disassembled states. CG31030 could then be important to facilitate the assembly of the two domains or maintain the assembled state, which may also influence neurotransmitter loading and release in a more continuous way. Our results suggest indeed that modulating V-ATPase activity in presynaptic terminals can finely affect quantal size. Whether such regulation actually occurs in physiological, or pathologic, conditions remains to be established.

Human *ATP6AP2*/*PRR* is known to be a Parkinsonism candidate gene and its mutations in humans, mice or flies can lead to cognitive impairment, neurodegeneration and epilepsy (Korvatska et al., 2013; Dubos et al., 2015; Ichihara and Yatabe, 2019). It is therefore of major interest to identify a potential new interactor of ATP6AP2 that is specific to the nervous system, as it could help better understand the consequences of V-ATPase dysregulation on synaptic transmission in pathologic contexts. Accordingly, it would be very interesting to determine whether human ATP6AP1 and/or ATP6AP1L share a conserved function with *Drosophila* CG31030 in the nervous system. Like CG31030, the mouse ATP6AP1L/AC45RP was recently reported to be restricted to neurons (Jansen et al., 2021), and they are, to date, the only known V-ATPase genes with such tissue specificity. Both proteins also have the particularity of not having a predicted furin cleavage site. ATP6AP1L/AC45RP was shown to be involved in neuronal growth (Jansen et al., 2021), while we observed that CG31030 downregulation also decreased the number of large synaptic boutons at the neuromuscular junction, hinting at a potential functional homology. Owing to its structural and functional similarity with its closest *Drosophila* homolog, VhaAC45/ATP6AP1, and vertebrates ATP6AP1L/AC45RP, we propose therefore that CG31030 be named VhaAC45-related protein (VhaAC45RP).

In conclusion, our present work identified a specific regulator of neuronal V-ATPase in *Drosophila* that is required for synaptic vesicle acidification, nervous system functioning and survival. Further study on this protein should provide a more detailed picture of the complex regulations surrounding neuronal V-ATPase specificity, revealing potential therapeutic targets and a better understanding of fundamental processes such as synaptic transmission.

## Synthesis

Reviewing Editor: Nathalie Ginovart, University of Geneva Decisions are customarily a result of the Reviewing Editor and the peer reviewers coming together and discussing their recommendations until a consensus is reached. When revisions are invited, a fact-based synthesis statement explaining their decision and outlining what is needed to prepare a revision will be listed below. The following reviewer(s) agreed to reveal their identity: Gerard Martens.

Reviewer 1:

The authors have done a reasonable job of addressing the queries; it is appreciated that there are limitations regarding reagents available in this model system.

Reviewer 2:

Points raised by reviewer #2 have been covered.

## Author Response

Point-by-point answers to Reviewers’ comments

We thank the Reviewing Editor and both Reviewers for their careful consideration of our manuscript eN-NWR-0193-21. We are very grateful for their positive appreciations and we believe that their suggestions, which have been included in the revised version of the manuscript, have greatly improved the quality of our work.

In their remarks, the two reviewers suggested additional experiments that could be done to improve our understanding of the role of this new V-ATPase regulator and raised some interesting issues. We have accordingly performed further experiments, whenever feasible, which have been included in the revision, and we have addressed all their comments. In our detailed answers below, the full text of the reviews is included in blue type, and the responses in black. The page and line numbers refer to the revised version of the manuscript, in which the main corresponding changes have been highlighted in yellow.

Please note that we have also followed the Instructions for Authors by inserting the Materials and Methods section immediately after the Introduction and removing any Supplemental material or mention of Supplemental material from the manuscript. As a consequence of this reorganization and the additional experiments, the tables have been renumbered, some figures have been modified and rebumbered, and new figures have been added.

Reviewer 1:

Here the authors present functional analysis of a new putative V-ATPase interacting protein in Drosophila. There continues to be a growing interest in V-ATPase function and regulation across many fields of biology; as such any new insights into modulatory mechanisms will be of interest.

Overall, the authors have explained each experimental step clearly and there is logic to the methods used to try and link new protein interaction data with downstream causation in physiological and behavioural outcomes. However, although the authors have been fair in their assessment of the data, in my opinion at least one or more of the main figures needs to be ‘tightened-up’ to support the conclusions:

1) Biochemistry: Can the interaction(s) be confirmed by standard IPs using the V5 tag? What is shown is only enrichment vs a non-tagged control IP and no specific co-localisation in tissue.

-- The product of the co-immunoprecipitation experiments could indeed be analyzed by western blot to confirm the results of mass spectrometry for some of the V-ATPase subunits. However, this method would require specific antibodies against each protein that we wish to test. Unfortunately, there are no commercially available antibodies against Drosophila V-ATPase proteins. To address the reviewer’s comment, we have tried several antibodies that recognize V-ATPase subunits from other species and were predicted to react against Drosophila homologs, but with no success. The only one currently at our disposal is a limited sample of a polyclonal antibody against Vha100-1, an isoform of V0 subunit a, which was generously sent to us by another laboratory. Thus, this technique would be of very limited application in our context. In contrast, mass-spectrometry analysis of the co-immunoprecipitation products presents two advantages: it has a higher sensitivity than western blots, and it allows unbiased analysis of the data since all Drosophila proteins from the reference databank are concomitantly being tested. Our results suggest that CG31030 interact, directly or indirectly, with V-ATPase subunits selectively located in the V0 domain. Interestingly, in a recent publication in mice (Jansen et al., Prog Neurobiol, 2021), the protein ATP6AP1L/AC45RP, which is a distant homolog of CG31030, was also shown to interact with the V-ATPase V0 domain, consistent with our findings. This is now mentioned page 21, lines 477-481 in the revised manuscript.

-- We also agree with the reviewer that some of the candidates identified by mass-spectrometry could correspond to proteins expressed in other cell types that interacted with CG31030 after tissue solubilization. However, this possibility seems very unlikely in the case of the V-ATPase because we identified three differents subunits of the V0 domain that co-immunoprecipitated with CG31030 with at least a two-fold abundance difference compared to the control in three independent experiments (Figure 4 and Table 3). As suggested by the reviewer, during the revision we have carried out a tissue co-localization experiment with one of these V0 domain protein, by co-immunostaining CG31030V5 and Vha100-1 (V0 subunit a) at the larval neuromuscular junction. As previously shown (Hiesinger et al., Cell, 2005), Vha100-1 is a subunit of the neuronal V-ATPase localized on synaptic vesicles. This experiment revealed that Vha100-1 and CG31030 co-localize at the Drosophila neuromuscular junction, confirming that they are expressed in the same cells and addressed to the same subcellular compartment. Results of this new experiment are shown in Figure 4C and D in the revised manuscript.

2) Localisation: Sub-fractionation and Western blotting would further support the co-localisation with the V-ATPase.

-- A sub-fractionation of celular components followed by a western blot could also be used to confirm that CG31030 and V-ATPase subunits are present in the same synaptic compartment. However, sub-fractionation of the Drosophila nervous tissues is not frequently done, as it is not very effective and requires large numbers of flies and special equipment. Furthermore, as mentioned in our answer to point 1 above, this technique would require sufficient quantities of antibodies against various Drosophila V-ATPase subunits that are not currently available. However, as stated in our answer above, we believe that the co-immunostaining of CG31030 and Vha100-1 at the neuromuscular junction now shown in the new Figure 4C and D provides a direct evidence that CG31030 and V-ATPase co-localize in nerve terminals.

3)Rescue / specificity: Can one of the key findings in the KD flies be rescued by gene replacement?

-- A rescue experiment could indeed potentially add further evidence that CG31030 deficiency is specifically responsible for the synaptic vesicle acidification defect (Figure 6), the decrease in locomotor performance (Figure 7) and the reduced quantal size (Figure 9). However, re-expressing CG31030 in the knockdown context using the UAS-Gal4 system would be difficult to interpret for the following reasons. If we observed a rescue in such experiment, we would have to control that the effect is not due to a possible decrease in RNAi expression due to co-expression of the CG31030 transcript. Conversely, if we did not observe a rescue in such experiment, we would have to control that the expressed CG31030 mRNA is not also significantly targeted by the RNAi, cancelling its potential rescuing effect. Because the expression with OK371-Gal4 is restricted to a subset of the fly neurons, such controls would not be possible by RT-qPCR. This is wwhy we chose to test the specificity of the phenotype by using two different RNAis, targeting distinct portions of the CG31030 transcript, as is currently done in such cases. Since we found that the observed phenotypes were similar with both RNAis, it is very unlikely that they could result from off-target effects.

4) Any changes in V-ATPase subunit protein expression levels?

-- We agree with the reviewer that it is a very interesting possibility, and we have carried out new experimentds to address this. Indeed, it has been recently shown in vitro that strong overexpression (about 7 folds) of mouse ATP6AP1L, a distant homolog of CG31030, led to increased transcript levels of several V-ATPase V0 domain subunits (Jansen et al., Prog Neurobiol, 2021). Here we have tested whether overexpression or partial knockdown of CG31030 could change the expression levels of other V0 domain subunits in Drosophila. Using pan-neuronal drivers, however, we were only able toincrease CG31030 transcript levels by 3.5 fold in vivo in wildtype context, suggesting that transcript abundance may be regulated post-transcriptionally, and this change had no effect on transcript levels of other V0 subunits, as shown in the new Figure 5A. We also performed RT-qPCR on heads of flies expressing CG31030RNAi3 in all neurons compared to controls. We did not find any significant change in expression levels of V0 subunits in knockdown context, and this result is also shown in the revised manuscript in Figure 5B. These experiments are described on page 21-22, lines 481-494, in the revised manuscript.

5) Any changes in V-ATPase assembly kinetics? or ATP hydrolysis?

-- Knowing whether the inactivation of CG31030 affects V-ATPase assembly kinetics or the efficiency rate of the proton pump would give us more insight on the precise function of CG31030 within the complex. Unfortunately, we could not find a suitable way to evaluate such mechanisms in vivo in Drosophila during this first study, and we consider that this issue would be better addressed in further work. Based on our results, our hypothesis is that CG31030 could be important in strengthening or maintaining the assembled complex, as stated in the Discussion section (lines 727-743).

6) There is a clear trend towards reduced frequency in the EPSPs in KD flies and quantal size can be influenced by many factors such as vesicle size / receptor dysfunction, etc. Are there any fundamental developmental or structural changes that may also explain these data?

-- The synaptic transmission defect we observed in knockdown larvae and the associated locomotor deficits could indeed be caused by other structural changes in the synapses, in addition to the problem of synaptic vesicle acidification. We have therefore performed new experiments to address this important point. To assess potential changes in synapse structure and function in CG31030 knockdown context, we checked two main parameters.

-- First we looked at the recycling of synaptic vesicles, as a defect in endocytosis or exocytosis could lead to a synaptic transmission decrease, potentially resulting in a locomotor impairment. For that, we carried out FM-dye loading/unloading experiments at the larval neuromuscular junction of knockdown animals versus controls. The results of these experiments are included in the revision in the new Figure 8 and described on lines 565-578. We observed that synapses of both knockdown larvae and controls were able to load and unload the dye similarly, and so no significant difference was observed in endocytosis or exocytosis levels. This suggests that CG31030 knockdown does not impact the synaptic vesicle release and recycling cycle, which is functioning normally and therefore could not be at the origin of the locomotor defect.

-- Secondly, as suggested by the reviewer, we have precisely examined the morphology of synapses at the neuromuscular junction of knockdown larvae compared to controls. This study showed that the overall number and mean size of synaptic boutons were not significantly affected between the different genotypes (new Figure 10, panels A-D). However, we observed a trend towards a reduced number of boutons with a diameter larger than 4 micrometers in the knockdown animals (Figure 10E and F). This new finding could be explained in several different ways. Neuronal activity is known to influence synaptic growth at the neuromuscular junction (Menon et al., Wiley Interdiscip Rev Dev Biol, 2013), so the lower amount of larger boutons in knockdown animals could be a consequence of the decreased synaptic transmission resulting from the synaptic vesicle acidification defect. As an alternative possibility, CG31030 downregulation could also partly hinders axonal growth as was recently shown for AC45RP/ATP6AP1L in mouse (Jansen et al., Prog Neurobiol, 2021).

In the revised manuscript, we had to take in account this last result for our interpretation of the quantal analysis experiment. Indeed, a lower number of high diameter boutons could contribute to the observed quantal size decrease, as larger boutons have been shown to harbor bigger synaptic vesicles associated with higher quantal content (Karunanithi et al., J Neurosci, 2002). The fact that these larger boutons only represent a small fraction of the total explains why the average number or mean size of boutons were not significantly affected in knockdown larvae. It is therefore unlikely that this size variation could result, by itself, in a significant decrease in quantal size. However, it is possible that this effect contributes, together with the synaptic vesicle acidification defect, to the observed effects on quantal size and locomotion. It could also explain the observed non-significant trend towards reduced frequency in the EPSPs of knockdown animals. This point is now stated in the revised manuscript (lines 598-613 and 754-756), and we are grateful to the reviewer for his constructive and very helpful suggestion.

Minor:

7) The authors are likely aware, but the vertebrate-specific ATP6AP1L protein has now been described in some detail (PMID: 33933532); thus this work warrants a citation and some discussion.

-- We did notice this recent article by Jansen et al. a few days after we submitted our manuscript to eNeuro as it was released about at that time. The reviewer is absolutely correct that we had to mention and discuss it in the revision. This article is now mentioned both in the Introduction (lines 74-77), Results (line 477-481) and Discussion (lines 751-756) sections. See also our answers to previous points above.

8) What is the working hypothesis regarding the biophysical properties/timing of V-ATPase V1/V0 assembly and the new data presented here?

-- It has been reported that the dissociation of the V1 domain from V0 on synaptic vesicles is a necessary step prior to exocytosis (Bodzęta et al., Cell Rep, 2017). When new vesicles are formed through endocytosis, the two domains then reassemble to start acidifying the vesicular lumen, allowing neurotransmitter uptake. Thus, during the synaptic vesicles recycling cycle, V0 and V1 dynamically alternate between assembled and disassembled states. We believe that CG31030 could be important to facilitate the assembly of the two domains or maintain the assembled state. This hypothesis is mentioned on lines 727-743 in the manuscript.

9) anti-V5 on control tissue should be shown

-- As requested, the control images have been added as the new Figure 3C and Figure 4D.

---

Reviewer 2:

Here authors study the role of a thus far unknown Drosophila protein CG31030 and identify it as an isoform of Drosophila Ac45, an accessory subunit of the Vacuolar-H-ATPase (V-ATPase). Its expression was shown to be neuronal and the study clearly points towards a role for this protein (denoted as VhaAC45-Like) in Drosophila development and neurotransmission. The manuscript clearly contributes to the field of V-ATPase regulation and neurotransmission. Below, you find my points of concern and suggestions that to my opinion would improve the manuscript prior to publication.

Point 1:

Title:

In what respect ‘specific’? What do the authors mean? Neural-specific? V-ATPase-specific? Drosophila-specific? Title should be changed in order to define ’specific’.

-- We thank rhe reviewer for this justified remark. The word “specific” refers here to the neuronal cell-type. The title has been changed to “A novel neuron-specific regulator of the V-ATPase in Drosophila” to avoid any confusion.

Point 2:

Introduction, lines 74 and 75: ATP6AP1L was only recently functionally characterized and termed Ac45-related protein (Ac45RP). It was shown to be brain-specific in vertebrates and involved in neuronal outgrowth (see Jansen et al. (2021)). To complete information regarding the Ac45-family, authors could also refer to the Ac45-like protein (Ac45LP), that was characterized in 2010 and was shown to be absent in placental mammals but replacing Ac45 in birds. Its expression in Xenopus was found to be kidney-and lung specific. (see Jansen et al. 2010). These combined data, including the present manuscript, show the diversity of V-ATPase regulation by Ac45 family members throughout the animal kingdom.

-- Thank you very much for pointing out these publications. They have been added in the Introduction (Lines 71-77) to complete the description of the current knowledge on the AC45 family, and also in the Discussion (lines 751-755). In light of the homology of CG3130 with vertebrate ATP6AP1L, and the recent functional characterization of the mouse ATP6AP1L that was named Ac45-related protein (AC45RP) (Jansen et al., 2021), we now accordingly propose to name CG31030 “VhaAC45RP” (lines 757-759).

Point 3:

Authors describe CG31030 as a single protein product. However, in the databases, two isoforms of the protein have been annotated: CG31030-PA (NP_733350.1) and CG31030-PB (NP_001036775.1) which differ in their C-termini. In this study, CG31030-PA is referred to as CG31030. This is of importance since functions of both proteins are unknown and it is not clear in the following RNAi experiments whether both transcripts are affected/downregulated. Could authors describe both variants and validate that both are downregulated by their RNAi approach?

-- Indeed, according to FlyBase, CG31030 encodes two predicted isoforms: CG31030-PA and CG31030-PB. The last 7 C-terminal amino-acids of CG31030-PA are theoretically replaced by a different segment of 46 amino-acids in CG31030-PB. Initially, we investigated the existence of these two isoforms and obtain preliminary data suggesting that CG31030-PB is actually not present in Drosophila. We have carried out further experiments during the revision that confirmed this result. Using primers framing the 55 bp difference between the corresponding predicted transcripts, we checked which isoform was expressed in Drosophila heads. Only a fragment corresponding to the expected size of CG31030-PA was amplified, suggesting that CG31030-PB was not expressed in this tissue. Consistent with this, PCR experiments using primers specific to CG31030-PB were not able to amplify anything, contrary to experiments using primers specific to CG31030-PA. These results are now shown in the new Figure 1A and B. Moreover, among the numerous cDNA clones matching CG31030 listed in FlyBase, either fully sequenced or only sequenced at the end (ESTs), all are consistent with CG31030-RA, and none specifically with CG31030-RB, supporting the idea that among the two predicted protein isoforms, only CG31030-PA is actually expressed in Drosophila. This is the reason why we refer to CG31030-PA simply as CG31030 throughout the paper. These data are now described in the Results section (lines 358-373) in the revised manuscript.

Point 4:

lines 139-141: Authors describe: Some neuronal cell bodies were also marked with the V5 antibody in the CG31030V5 line, both in adult and larva, and the synapse-containing neuropile areas (like the antennal lobes) were not as sharply defined as with the CadN antibody, suggesting that axons could also be immunopositive.

What do the authors mean? Vague description of their results, please rephrase and point out and indicate in Figure 5.

Also: Figure 5D: what do the green-fluorescents regions that do not stain for CadN represent?

-- Figure 3 displays the co-staining of V5-tagged CG31030 and the synaptic marker CadN. The two patterns are similar as they both strongly mark synaptic areas. However, all cell bodies are also faintly marked by the anti-V5, corresponding to the green-fluorescent regions that are not co-stained with CadN. Among the cell bodies, a few appear as strongly marked as the synapses by the anti-V5 antibody (arrowheads have been added to point them out in Figure 3A and B. At this stage, we don’t know why a few neurons apparently contain a higher level of CG31030 in the cell bodies compared to most other neurons of the CNS. To address the legitimate questions of the reviewer, these images are now better described in the main text (lines 437-446) and the legend to Figure 3.

Point 5:

Some additional biochemical characterization of the new Ac45 family members would be appreciated. For instance, ProP 1.0 propeptide cleavage site prediction predicts proVhaAC45 cleavage by furin at position 185 (RRTRR/DT) which resembles the mammalian cleavage site (see Louagi et al. (2008)). Cleavage of Ac45 is known to be crucial for its intracellular trafficking and functioning (Jansen et al. (2008, 2010), Louagi et al. 2008). On the other hand, the same prediction program does not indicate any cleavage site in the CG31030 protein, pointing to constitutive action of this protein towards the V-ATPase. Western blot analysis using the V5 epitope should reveal its molecular weight and show whether CG31030 is proteolytically cleaved, like Ac45 or not (like Ac45LP and Ac45RP). Furthermore, deglycosylation experiments might show whether the protein is post-translationally modified by the addition of glycans, a characteristic of all Ac45-family members thus far described. Alignment in supplementary figure 1: indicate predicted furin-cleavage site in VhaAC45 at position 185.

-- As suggested by the reviewer, we have added the predicted cleavage site of VhaAC45 (which is also present in ATP6AP1/AC45) in the structural comparison of Figure 1C of the revised manuscript, to highlight the difference with CG31030 which has indeed no predicted cleavage site, as is the case for ATP6AP1L/AC45RP,. According to the NetNGlyc 1.0 server, Drosophila VhaAC45 has 3 N-glycosylation sites while CG31030 has only 2. These sites are now also indicated in Figure 1C. In addition, we have highlighted the transmembrane domains and the conserved pair of cysteine residues on both proteins to better represent their molecular structure.

-- As suggested, we have also performed a western blot on CG31030-V5 and control samples to determine the size of the protein (new Figure 1E). Two distinct bands, close to each other, were detected specifically in the CG31030-V5 sample, consistent with the presence of glycosylated and unglycosylated forms of the protein. While the theoretical size of CG31030-V5 is 44 kDa, the observed bands migrated at about 32 kDa as compared to a molecular weight marker. This could indicate that the protein is actually cleaved, like its paralog VhaAC45, despite the absence of a predicted cleavage site. However, it is known that many membrane proteins migrate faster than water-soluble proteins on SDS gels, leading to an underestimation of their size (Rath et al., PNAS, 2009). This could also explain the lower apparent size of CG31030 in western blots. As a result of these changes, the predicted structure of CG31030 is now more accurately described in the revised manuscript (lines 378-381 and 384-392).

Point 6:

Authors show direct or indirect interactions between CG31030 and, among other unknown proteins, V-ATPase subunits. This raises the question whether CG31030 downregulation affects the endogenous expression of V0 subunits, in particular ATP6AP2 and Ac45. Is Ac45 still expressed in knockdowns cells? Can it substitute CG31030 protein?

-- It was indeed very interesting to determine whether CG31030 downregulation could affect the expression of V0 subunits, as it was recently shown that overexpression of AC45RP/ATP6AP1L increases transcript abundance of these genes (Jansen et al., Prog Neurobiol, 2021). To assess this, we performed RT-qPCR on extracts of Drosophila heads expressing CG31030RNAi.3 in all neurons compared to controls. We did not find any significant change in the level of expression of V0 subunits (including ATP6AP2 and VhaAC45) in knockdown context, and this figure has been added to the paper (new Figure 5B). It should noted that VhaAC45 is expressed in cells of CG31030 knockdown flies at a similar level as in controls. The lethal phenotype and the highly reduced longevity occurring when CG31030RNAi.1/2 or CG31030RNAi.3 are expressed in all neurons, respectively, suggest that VhaAC45, though still present, is unable to substitute for CG31030. This is now mentioned in the manuscript on lines 490-494.

Point 7:

Figure 5E: what was the n in this experiment? Could significance be reached by increasing the number of experiments? In this way a difference in the number of vesicles released and the amount of neurotransmitter per vesicle could be determined and a more precise conclusion could be drawn.

-- The recorded signals used for the frequency analysis are the same as the ones used for the quantal analysis, meaning 6-7 cells per genotype. This n value has now been added in the legend to Figure 9, which corresponds to the electrophysiology experiments, according to the new figure numbering. We agree with the reviewer that increasing the n could possibly result in a significant difference in the frequency analysis. However, we did not have the possibilty to repeat these experiments again for contextual reasons, and we found nevertheless very interesting that the same n value was sufficient to measure a statistically-significant difference in quantal size but only a trend towards reduced frequency in the same knockdown animals. This potentially revealed a higher variability in frequency that could be the result of a less penetrant phenotype induced by CG31030 knockdown, possibly related to its subtle effect on the neuromuscular junction morphology (for more details about that and the corresponding changes we introduced in the manuscript, please see our answer to Point 6 of the Reviewer 1 above).

Line 339 Typo: increased

line 523: Typo: CRISPR/Cas9 tagging

-- These two typos have been corrected.
