# Erratum: Dulac et al., “A Novel Neuron-Specific Regulator of the V-ATPase in *Drosophila*”

**DOI:** 10.1523/ENEURO.0389-22.2022

**Published:** 2022-10-11

**Authors:** Amina Dulac, Abdul-Raouf Issa, Jun Sun, Giorgio Matassi, Célia Jonas, Zohra Rahmani, Baya Chérif-Zahar, Daniel Cattaert, Serge Birman

**Affiliations:** 1 Genes Circuits Rhythms and Neuropathology, Brain Plasticity Unit, Unité Mixte de Recherche 8249, Centre National de la Recherche Scientifique, École Supérieure de Physique et de Chimie Industrielles de la Ville de Paris, Paris Sciences et Lettres University, Paris F-75005, France; 2 Dipartimento di Scienze Agroalimentari, Ambientali e Animali, University of Udine, Udine I-33100, Italy; 3 Centre National de la Recherche Scientifique, Institut de Neurosciences Cognitives et Intégratives d’Aquitaine, Unité mixte de Recherche 5287, Université de Bordeaux, Bordeaux F-33000, France; 4 Centre National de la Recherche Scientifique, Unité Mixte de Recherche 7058 “Ecologie et Dynamique des Systèmes Anthropisés” (EDYSAN), Université de Picardie Jules Verne, Amiens cedex F-80025, France

In the article “A Novel Neuron-Specific Regulator of the V-ATPase in *Drosophila*,” by Amina Dulac, Abdul-Raouf Issa, Jun Sun, Giorgio Matassi, Célia Jonas, Baya Chérif-Zahar, Daniel Cattaert, and Serge Birman, which published online on October 7, 2021, an author was inadvertently omitted from the author line. Zohra Rahmani should appear sixth and be credited with performing research and analyzing data. The article has been updated online, and the corrected author line, affiliations, and contribution statement are included below.

Author contributions: A.D., D.C., and S.B. designed research; A.D., A.-R.I., J.S., G.M., C.J., Z.R., B.C.-Z., D.C., and S.B. performed research and analyzed data; A.D. and S.B. wrote the paper with input from all authors.

